# Supplementary figures and images for: The impact of age on genetic risk for common diseases
Source: PLoS Genet. 2021 Aug 26;17(8):e1009723. doi: 10.1371/journal.pgen.1009723 (PMC8389405; doi:10.1371/journal.pgen.1009723)

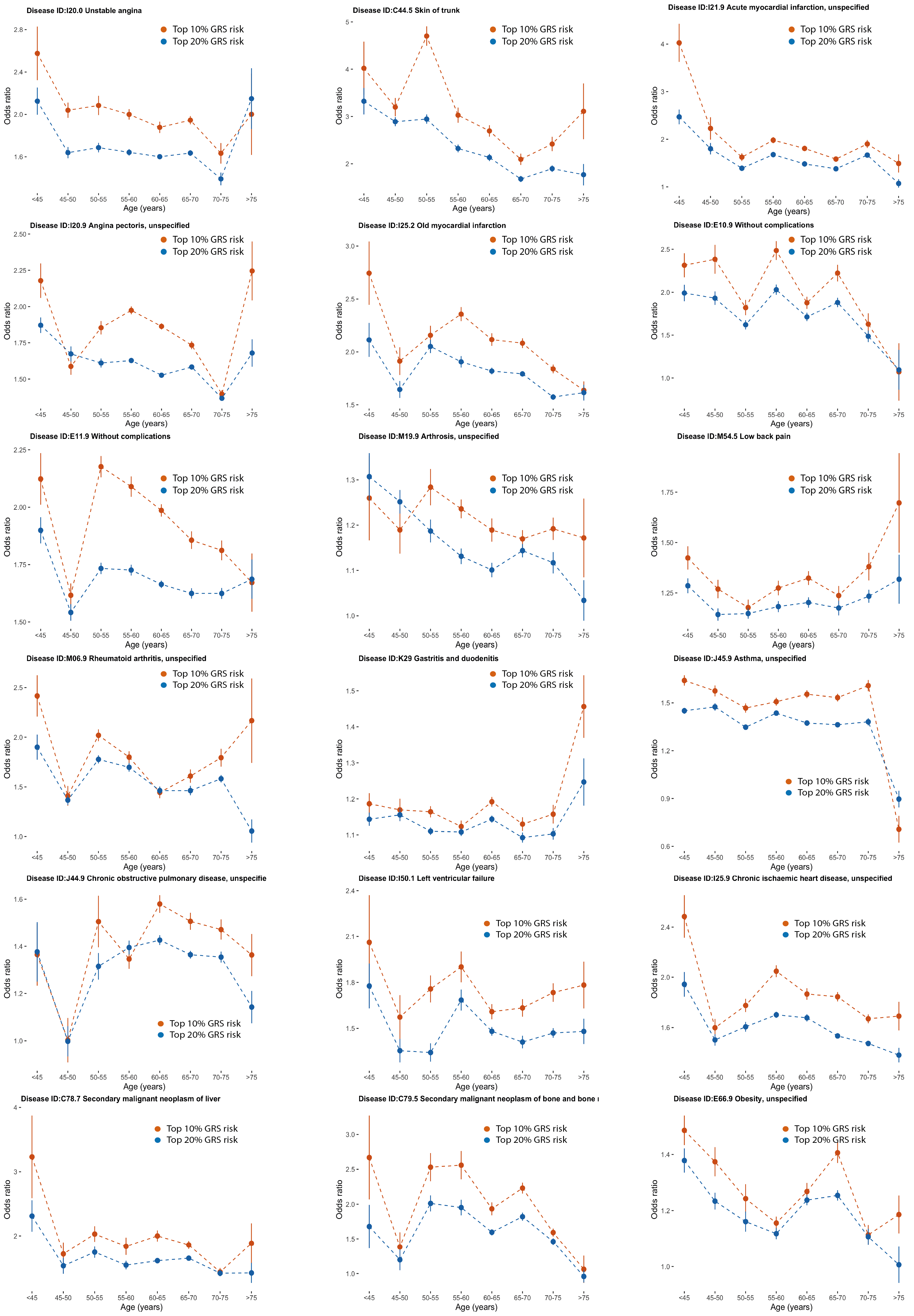

Supplement: S1 Fig — In each plot the odds ratios for the 80th (blue) and 90th percentiles of a combined genetic risk score within matched case-control samples (four controls for each case) are shown for each age interval; points indicate the average odds ratio of twenty five-fold cross-validation analyses with lines indicating the 95% confidence interval. (PNG) [file pgen.1009723.s003.png]

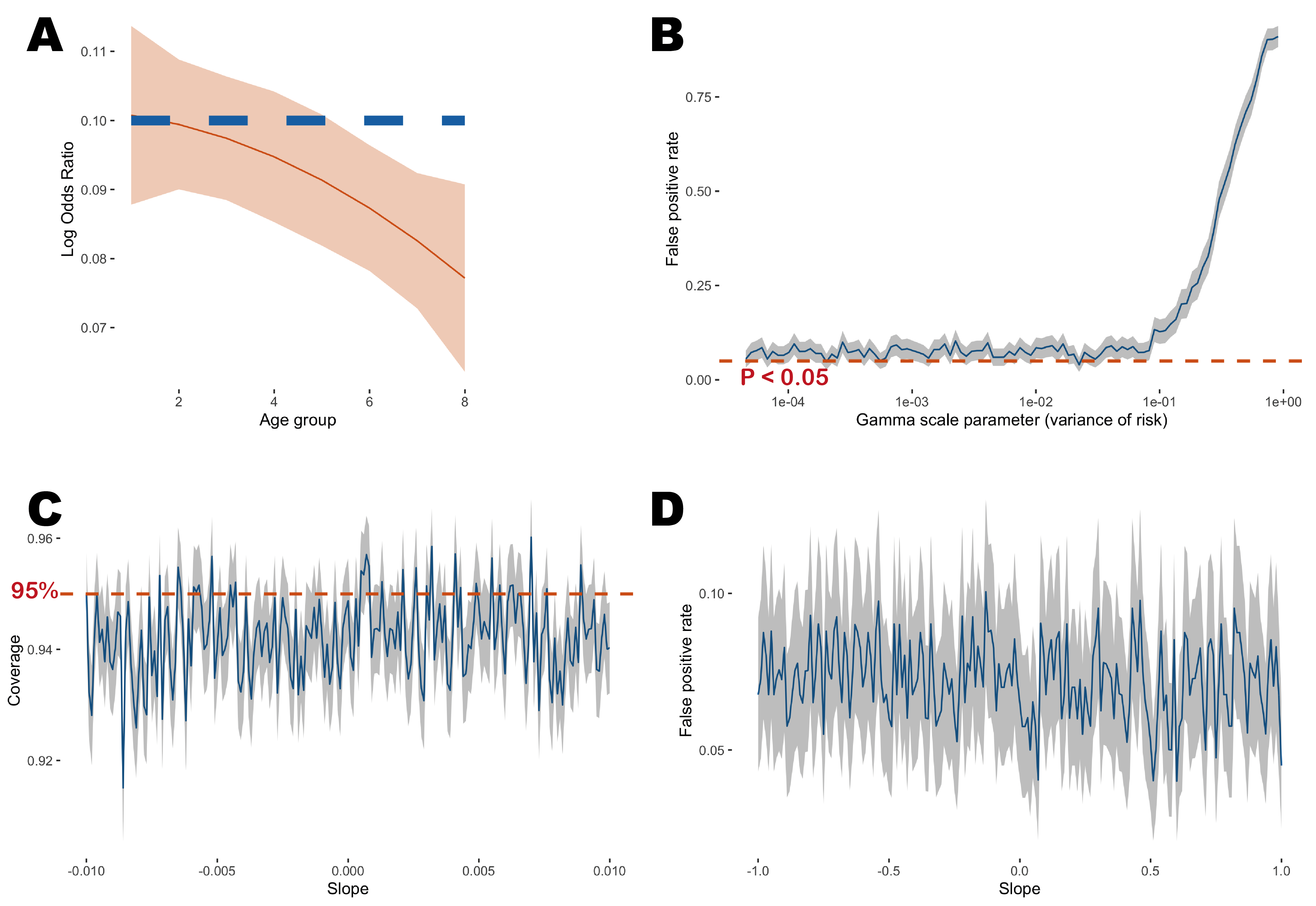

Supplement: S2 Fig — (A) A simulation with frailty showing that the inferred effect (red) deviates from the underlying effect size (blue dashed line). The variance of frailty in this case is 0.82. (B) Effect of frailty on the false positive rate. The x-axis shows the variance of the frailty distribution, with a larger variance indicating stronger frailty, while the y-axis is the false positive rate of rejecting the true model of constant effect over age. The inferred curve does not deviate from uniformity when the frailty variance is smaller than 0.1. (C) Coverage analysis. The blue curve shows the probability that 95% posterior credible interval covers the true genetic profile and the shaded area is the 95% confidence interval of the coverage estimate. (D) Simulation to test the impact of selecting healthier individuals of older age. Selection bias towards healthier older people is simulated by changing the baseline hazard over age, such that a negative slope indicates a population in which older people are biased away from having disease. The blue solid line shows the false positive rate of rejecting the null hypothesis of uniformity for a baseline hazard with different slopes; the shaded area shows the 95% confidence interval. Genetic profile estimation uses a quadratic polynomial throughout. (PNG) [file pgen.1009723.s004.png]

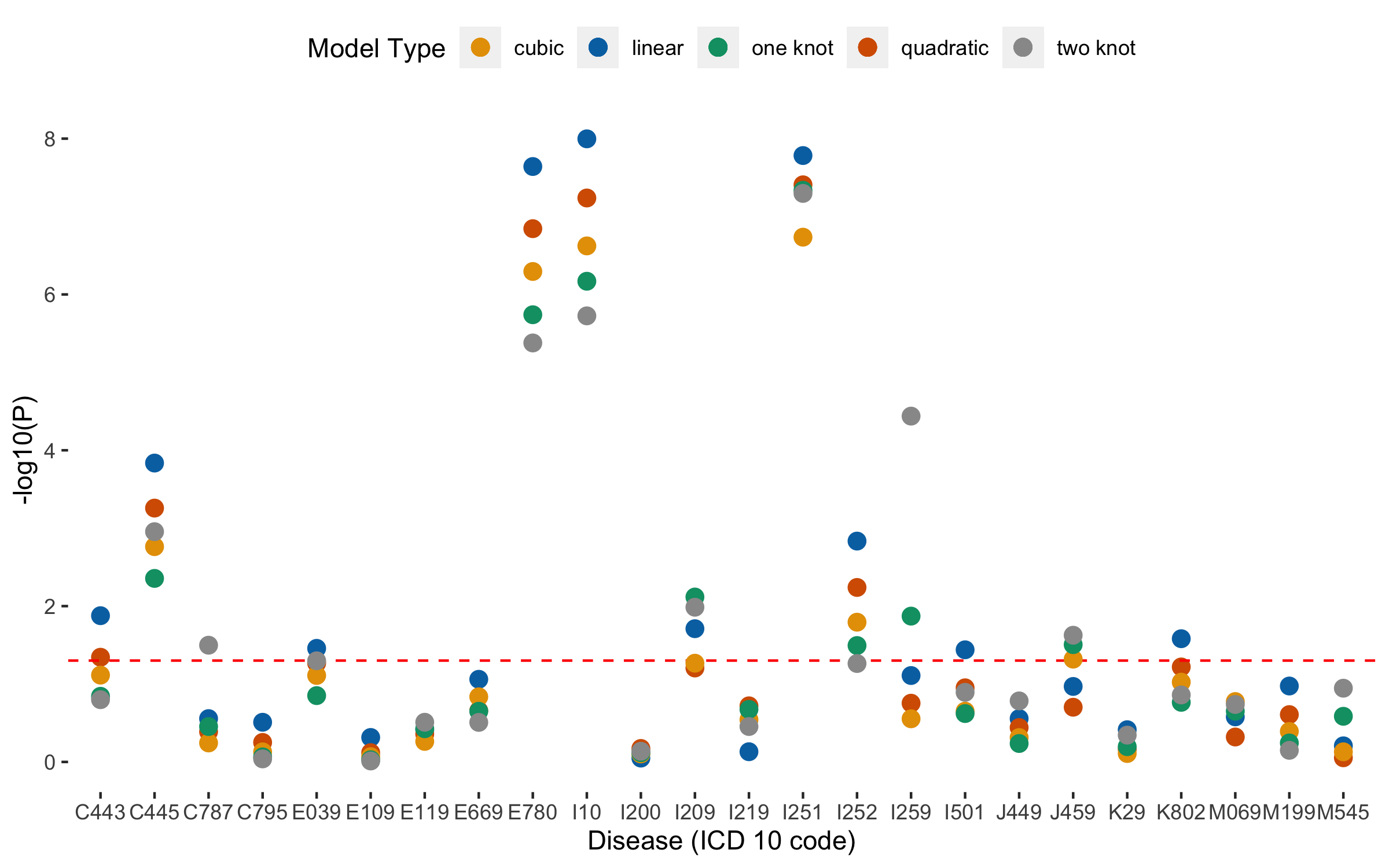

Supplement: S3 Fig — A likelihood ratio test is performed against a constant effect model (DF = 1) over age, for models with different smoothness. Smoothness is controlled by the degree of freedom of the spline basis, where we tested linear (DF = 2, blue), quadratic polynomial (DF = 3, red), cubic polynomial (DF = 4, orange), spline with one knot (DF = 5, green) and spline with two knots (DF = 6, grey). The red dotted line indicates P = 0.05. (PNG) [file pgen.1009723.s005.png]

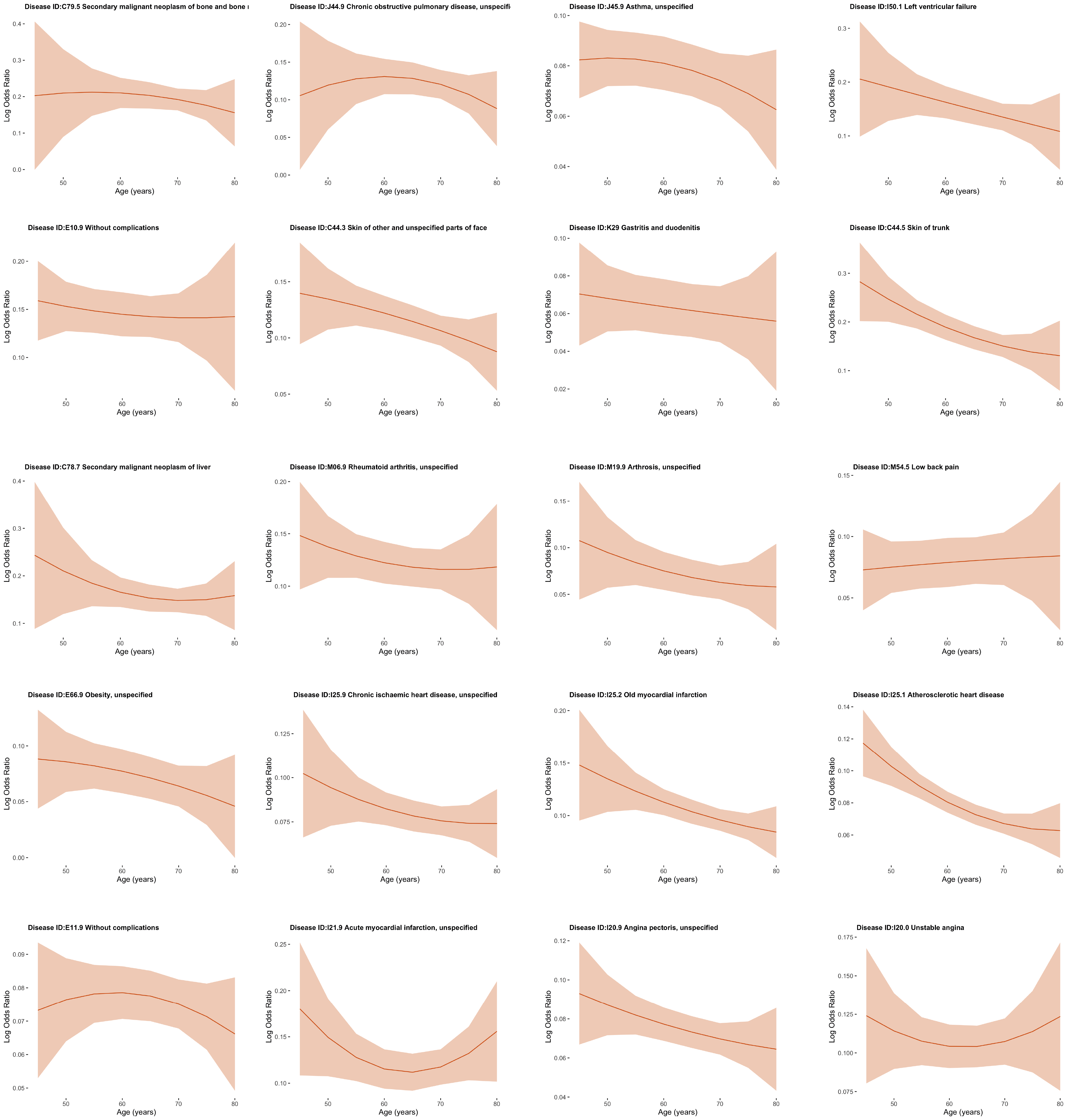

Supplement: S4 Fig — The solid red curve indicates the posterior mean, and the shaded region is the 95% credible interval. (PNG) [file pgen.1009723.s006.png]

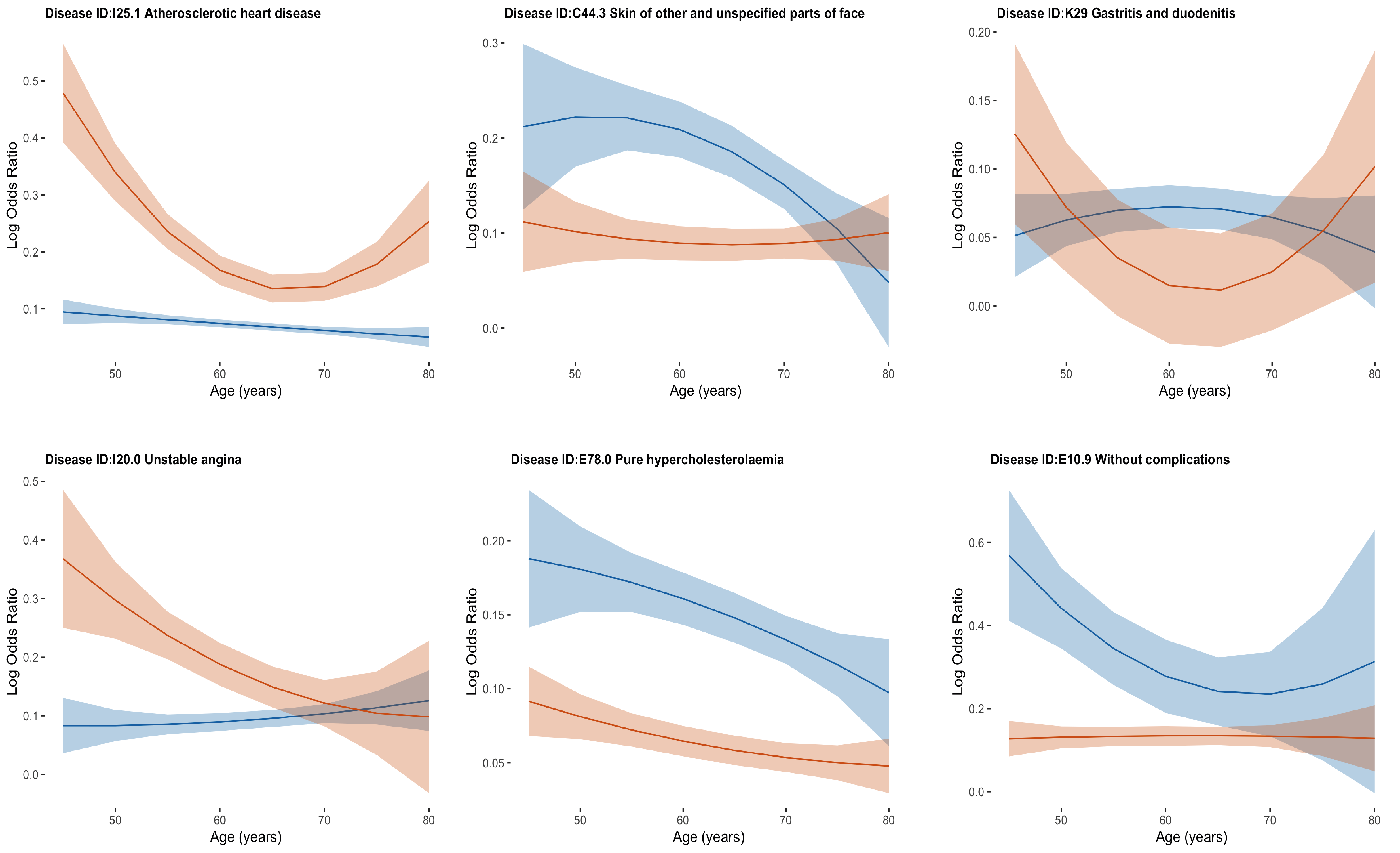

Supplement: S5 Fig — The red and blue curves with corresponding shades show the profile means and 95% credible intervals for each profile. (PNG) [file pgen.1009723.s007.png]

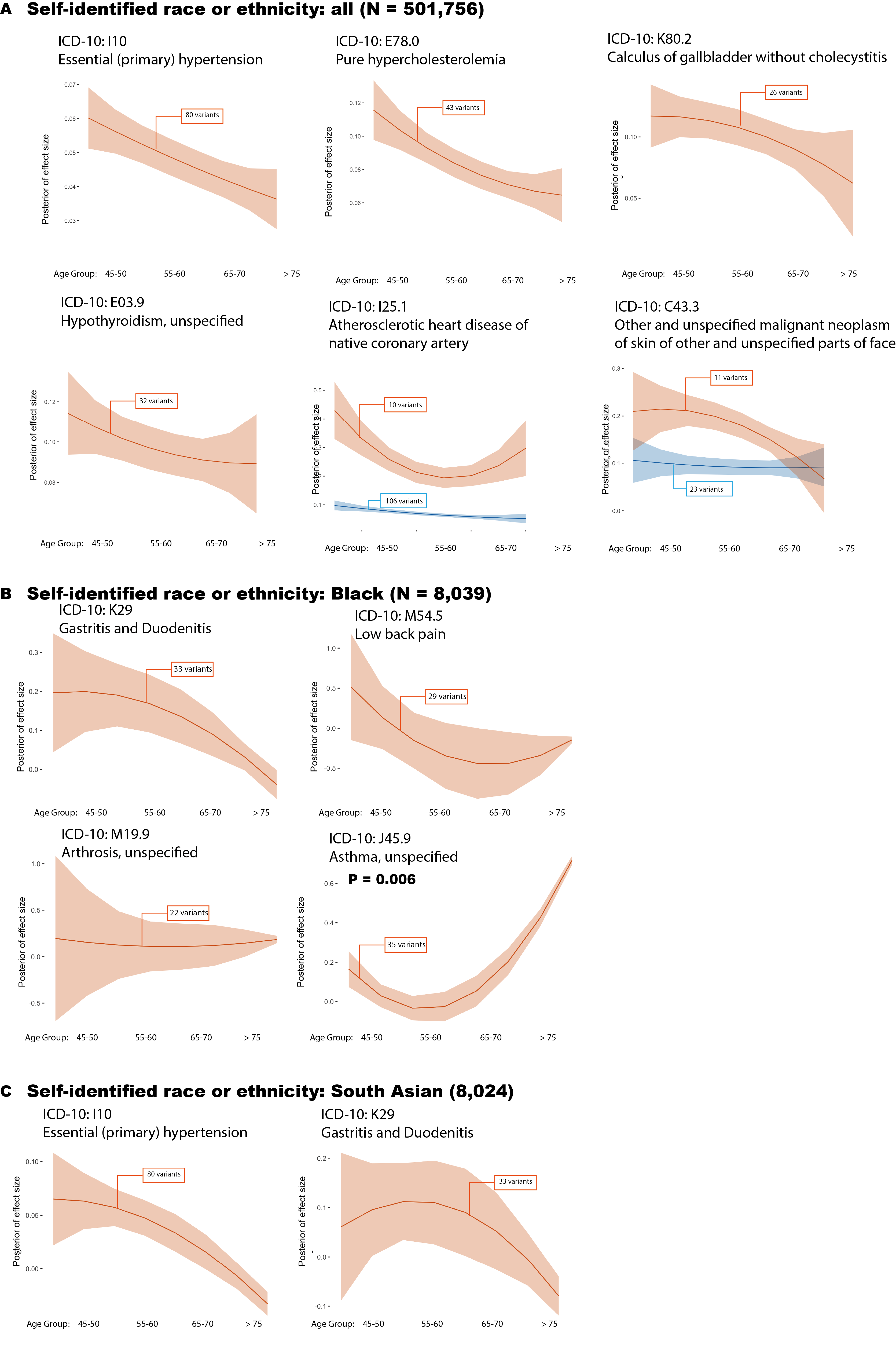

Supplement: S6 Fig — (A) Inferred cluster profiles for the six disorders presented in Fig 4 using all subjects in UK Biobank regardless of ethnicity. The blue and red curves in the last two figures indicate the means for the two clusters of variants, where there is evidence for multiple profiles within the British Isle ancestry group. Curves for all diseases are shown in S7 Fig. (B) Inferred cluster profiles for individuals self-identified as Black ethnicity in the UK Biobank. “Gastritis and duodenitis” (ICD-10 code K29), “low back pain” (M54.5), “Arthrosis” (M19.9) and “Asthma” (J45.9, P = 0.006). We also include the permutation P-value for asthma as its age profile has a uniquely increasing risk profile. (C) Inferred cluster profiles for individuals self-identified as South Asian ethnicity; “primary (essential) hypertension” (I10) and “Gastritis and duodenitis” (K29). The solid line indicates the posterior mean and the shaded area the 95% credible interval; Numbers in boxes indicate the number of variants in each cluster; All estimates are made with quadratic models for age-varying risk profiles. (PNG) [file pgen.1009723.s008.png]

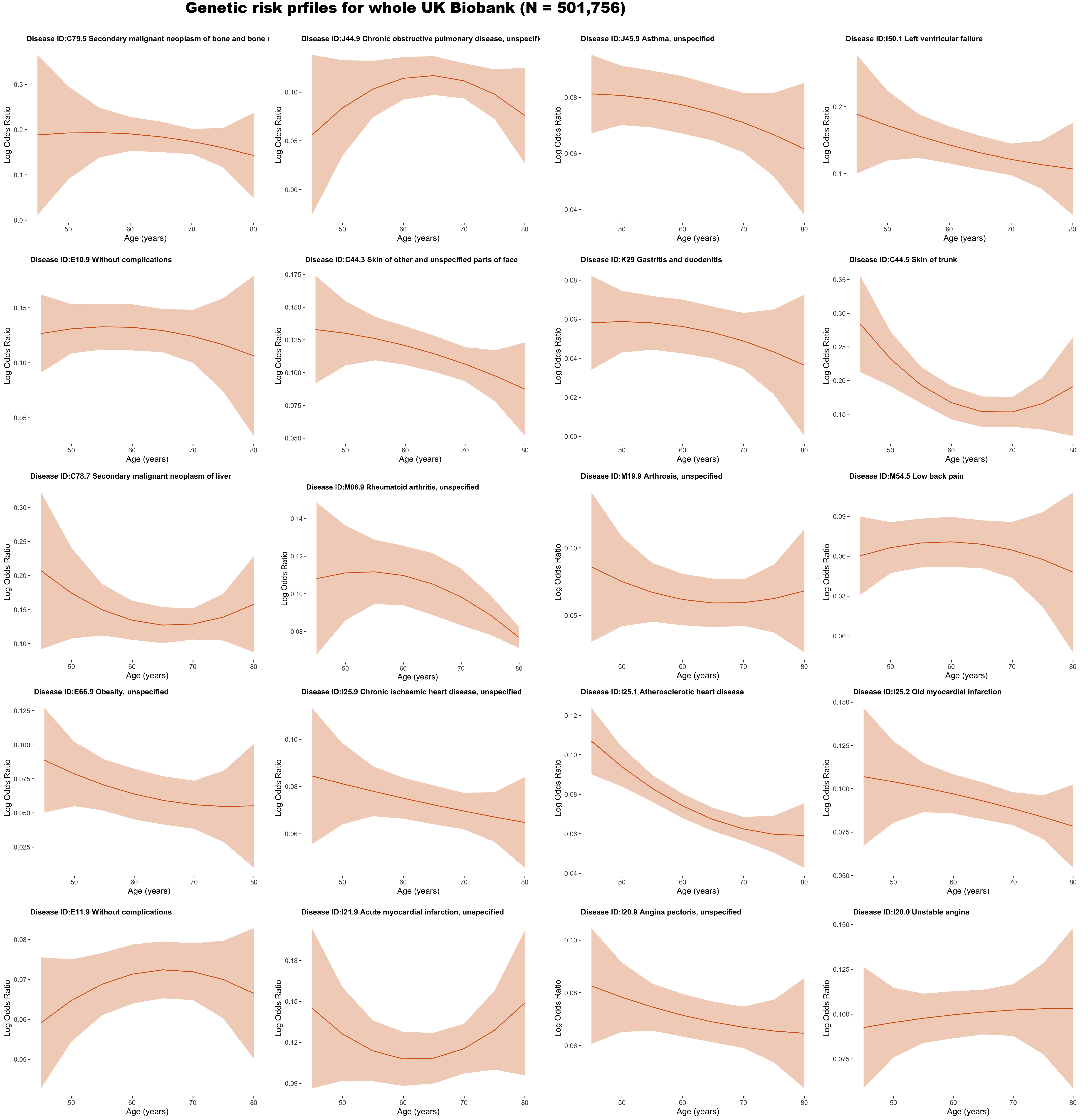

Supplement: S7 Fig — The solid red curve indicates the posterior mean, and the shaded region is the 95% credible interval. (PNG) [file pgen.1009723.s009.png]

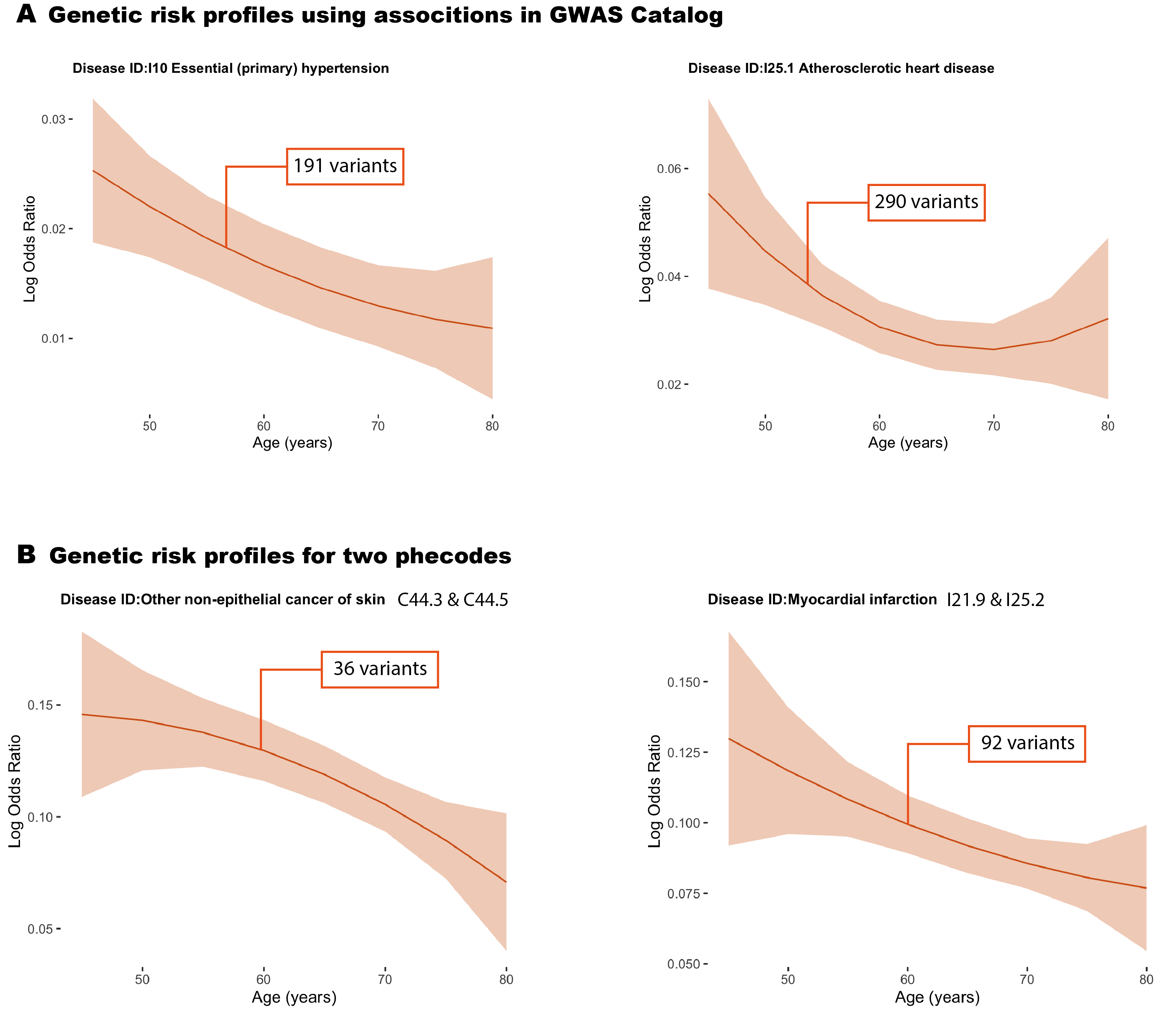

Supplement: S8 Fig — (A) Inferred cluster profiles using associations collected from GWAS Catalog for two disorders: “primary (essential) hypertension” (I10) and “antherosclerotic hearth disease” (I25.1). (B) Inferred cluster profiles for two phecodes: “172.20” (other non-epithelial cancer of skin, ICD-10 codes: C44.3 and C44.5) and “411.20” (myocardial infarction, ICD-10 codes: I21.9 & I25.2). The solid line indicates the posterior mean and the shaded area the 95% credible interval; Numbers in boxes indicate the number of variants in each cluster; All estimates are made with quadratic models for age-varying risk profiles. (PNG) [file pgen.1009723.s010.png]

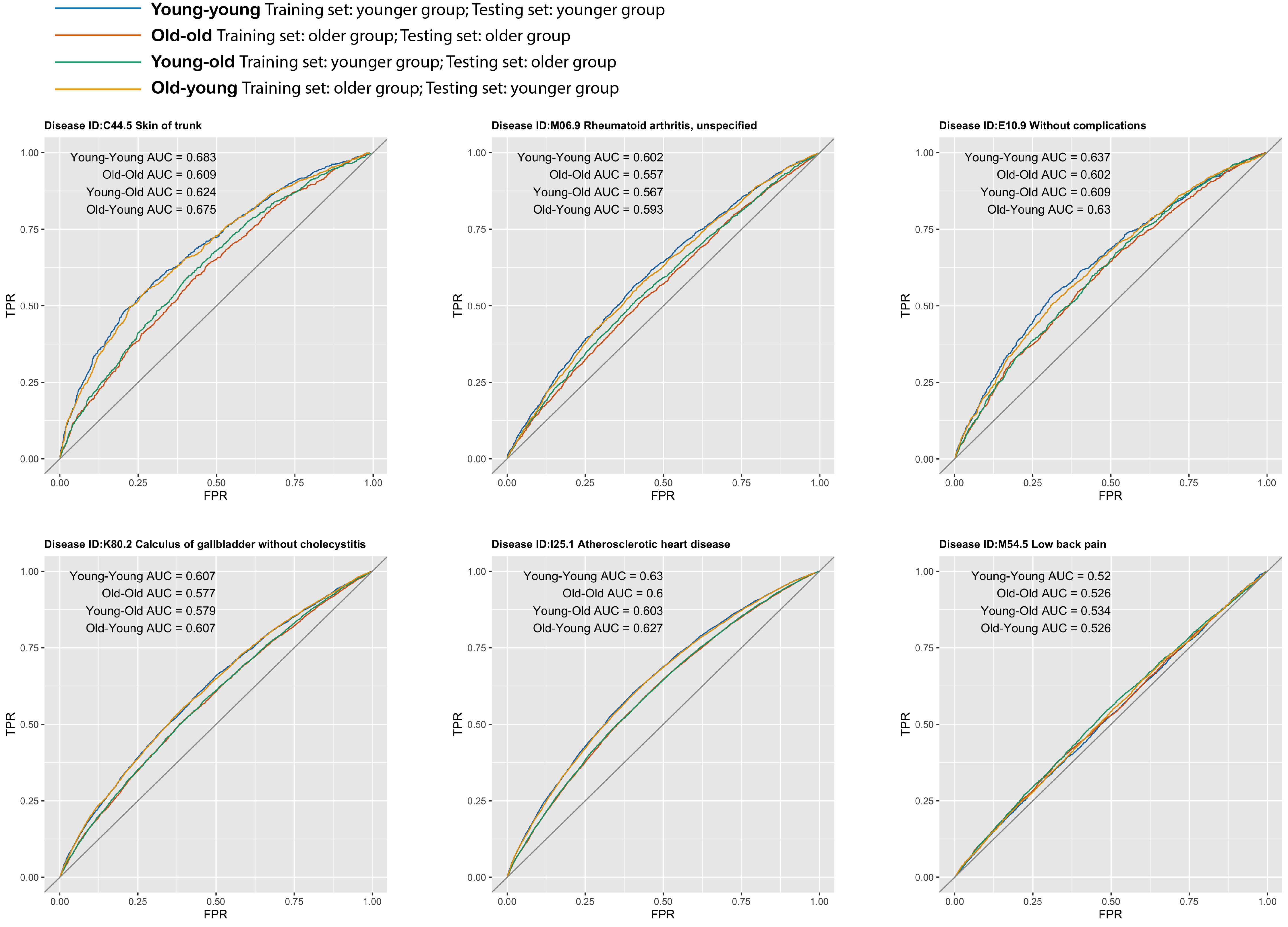

Supplement: S9 Fig — The population is divided into a younger group and an older group, where GRS and ROC are estimated from one of the groups. Each plot shows the ROC for four combinations: GRS and ROC are both computed from younger (Young-young; blue) or older group (Old-old; red); GRS is computed from the younger group and ROC is computed from the older group (Young-old; green) and the other way around (Old-young; orange). The area under the curve (AUC) metrics are shown in the top left. C44.5 “other and unspecified malignant neoplasm of skin of trunk”, M06.9 “rheumatoid arthritis, unspecified”, E10.9 “type 1 diabetes mellitus without complications”, K80.2 “calculus of gallbladder without cholecystitis”, and I25.1 “atherosclerotic heart disease of native coronary artery” are ICD-10 codes that have the biggest AUC differences between the blue and red ROCs. M54.5 “low back pain” is the only disease that has a larger AUC in red ROC than blue ROC. Regardless of whether the GRS and ROCs are computed from the same age group, we use 80% of the sample to compute the GRS and 20% for the ROC to match the sample sizes under each condition. (PNG) [file pgen.1009723.s011.png]

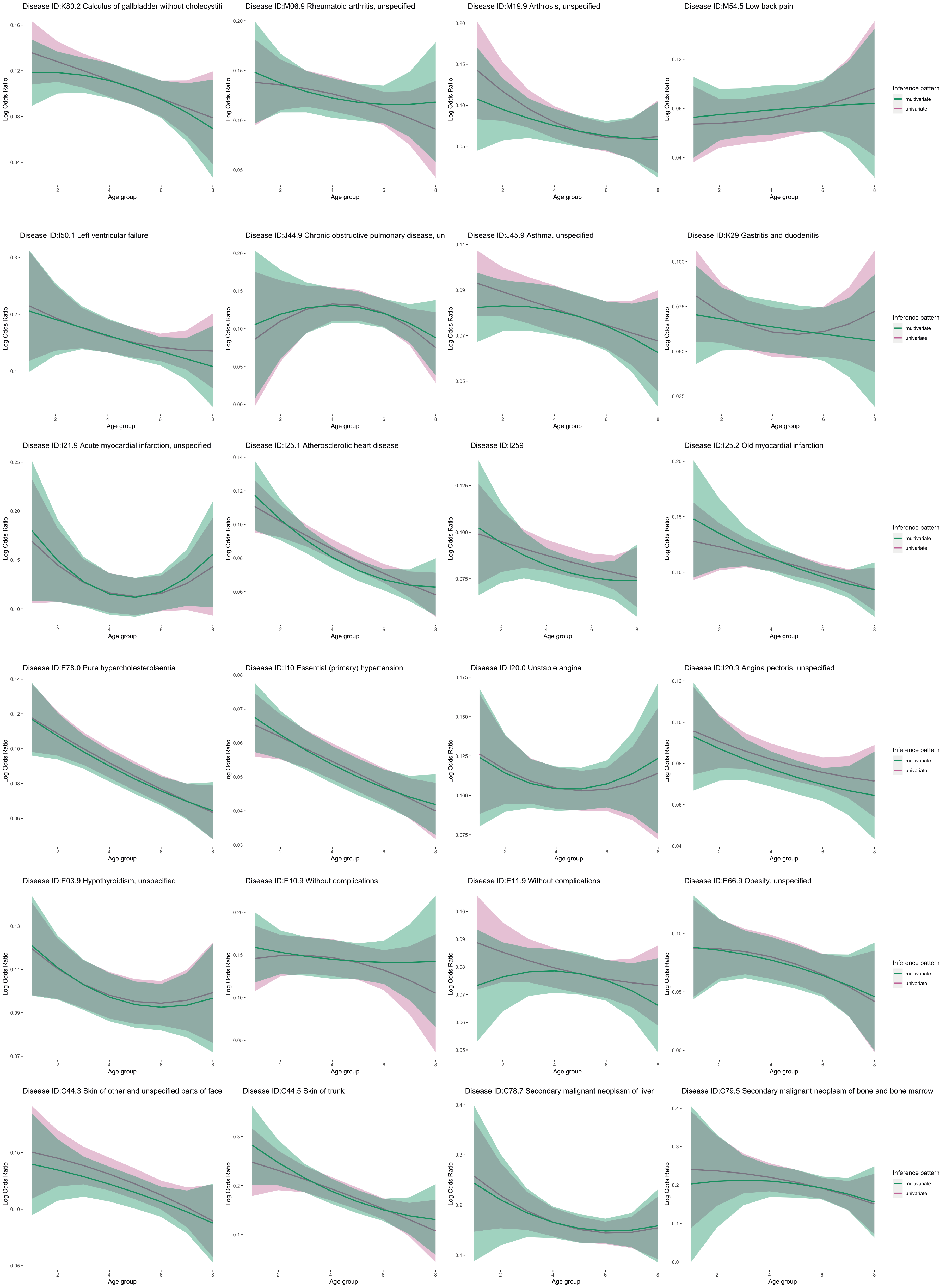

Supplement: S10 Fig — A quadratic polynomial model is fitted to the estimated effect size in both cases, which is shown as two curves: green (multivariable) and purple (univariable). (PNG) [file pgen.1009723.s012.png]

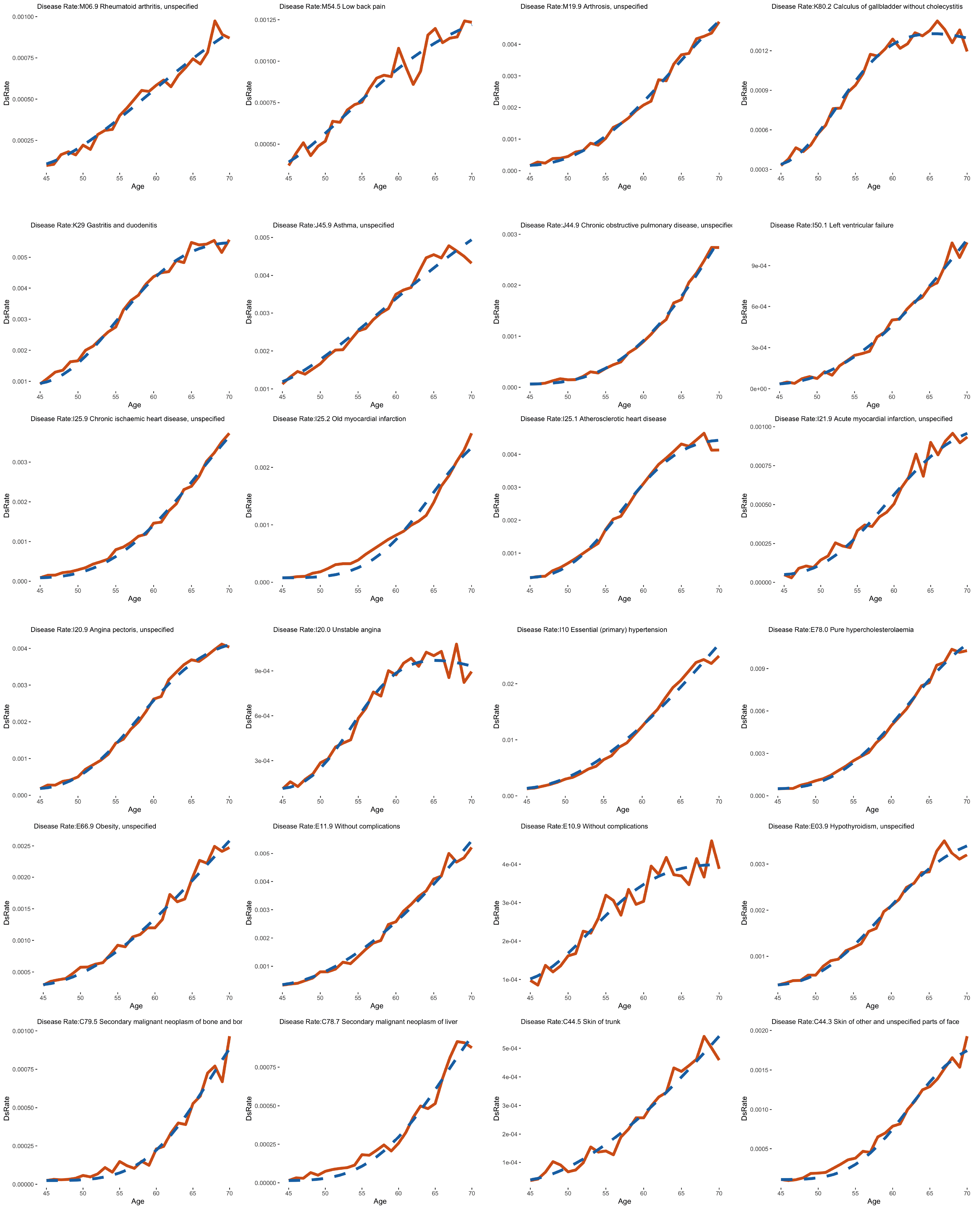

Supplement: S11 Fig — The red solid line indicates the rate estimated from the UK Biobank (see S1 Supplemental Methods) and the dotted blue line indicates the fitted incidence curve from the parametric model. (PNG) [file pgen.1009723.s013.png]

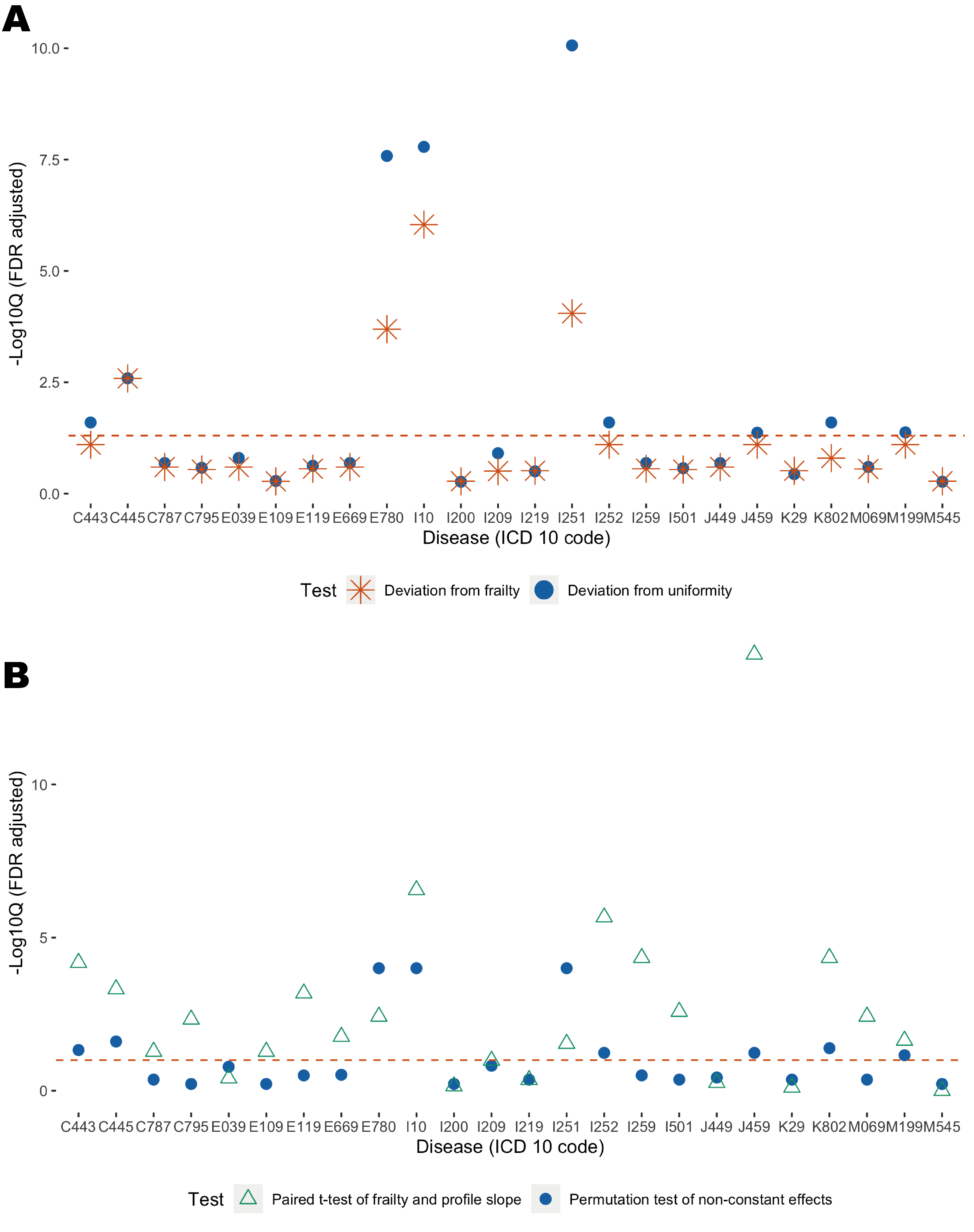

Supplement: S12 Fig — A) A likelihood ratio test of deviation from the fitted frailty model (red), compared with the likelihood ratio test of deviation from a constant effect model. Four diseases have Q < 0.05 after correcting for multiple testing. All inferences are performed on the univariable estimation of variant effect size because the fitted frailty should include both genetic and non-genetic factors. B) Paired t-test of the gradient of frailty and our inferred curve, identifying 17 out of the 24 diseases analysed where the inferred genetic risk profile slope is steeper than that implied by the inferred frailty parameters. (PNG) [file pgen.1009723.s014.png]

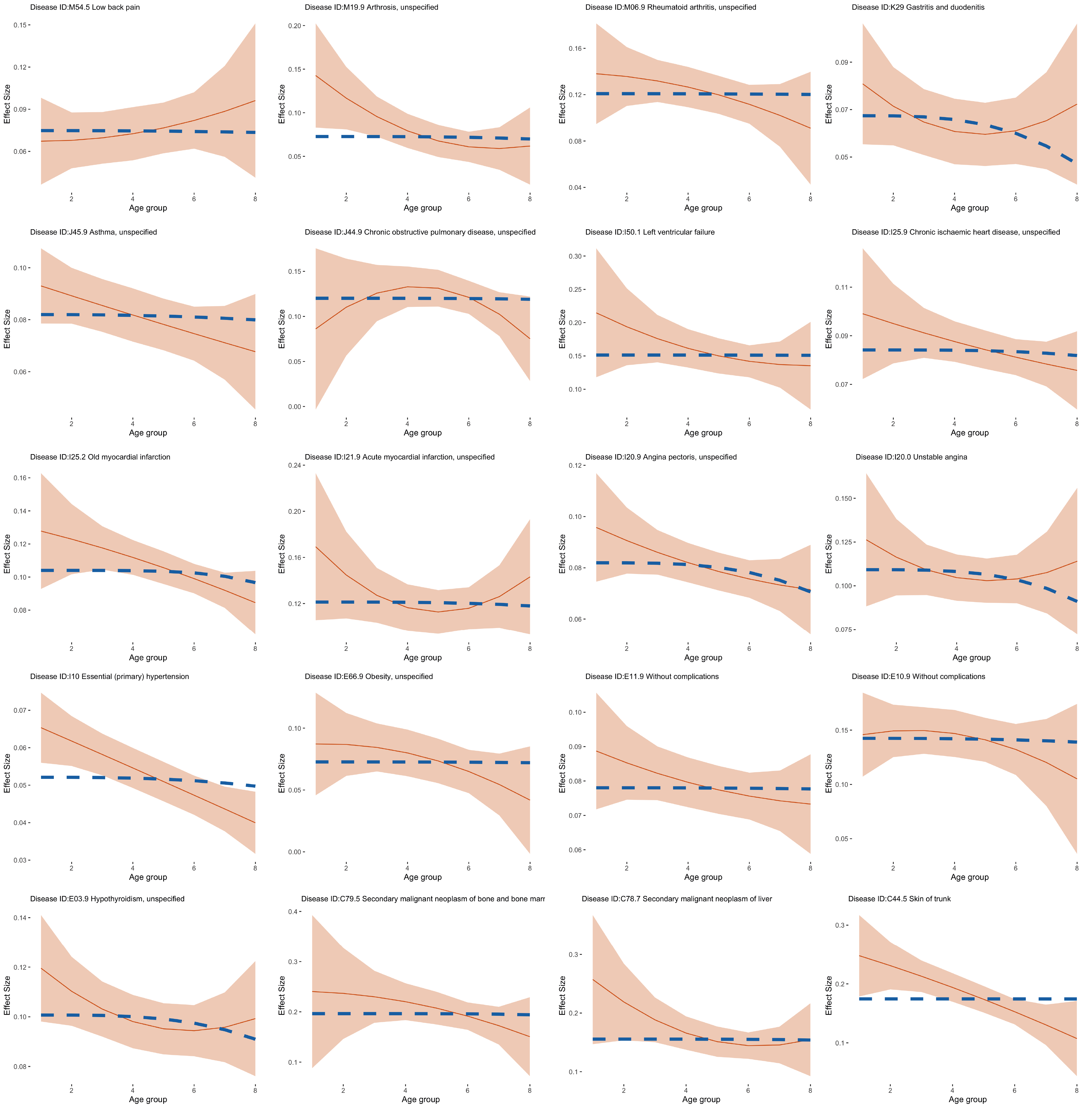

Supplement: S13 Fig — Comparison of fitted latent curves (red curve for the mean and shaded region for the 95% credible interval, estimated using the univariable approach) and latent profiles implied by the fitted frailty effect (blue dashed line), for all 24 diseases analysed here. (PNG) [file pgen.1009723.s015.png]

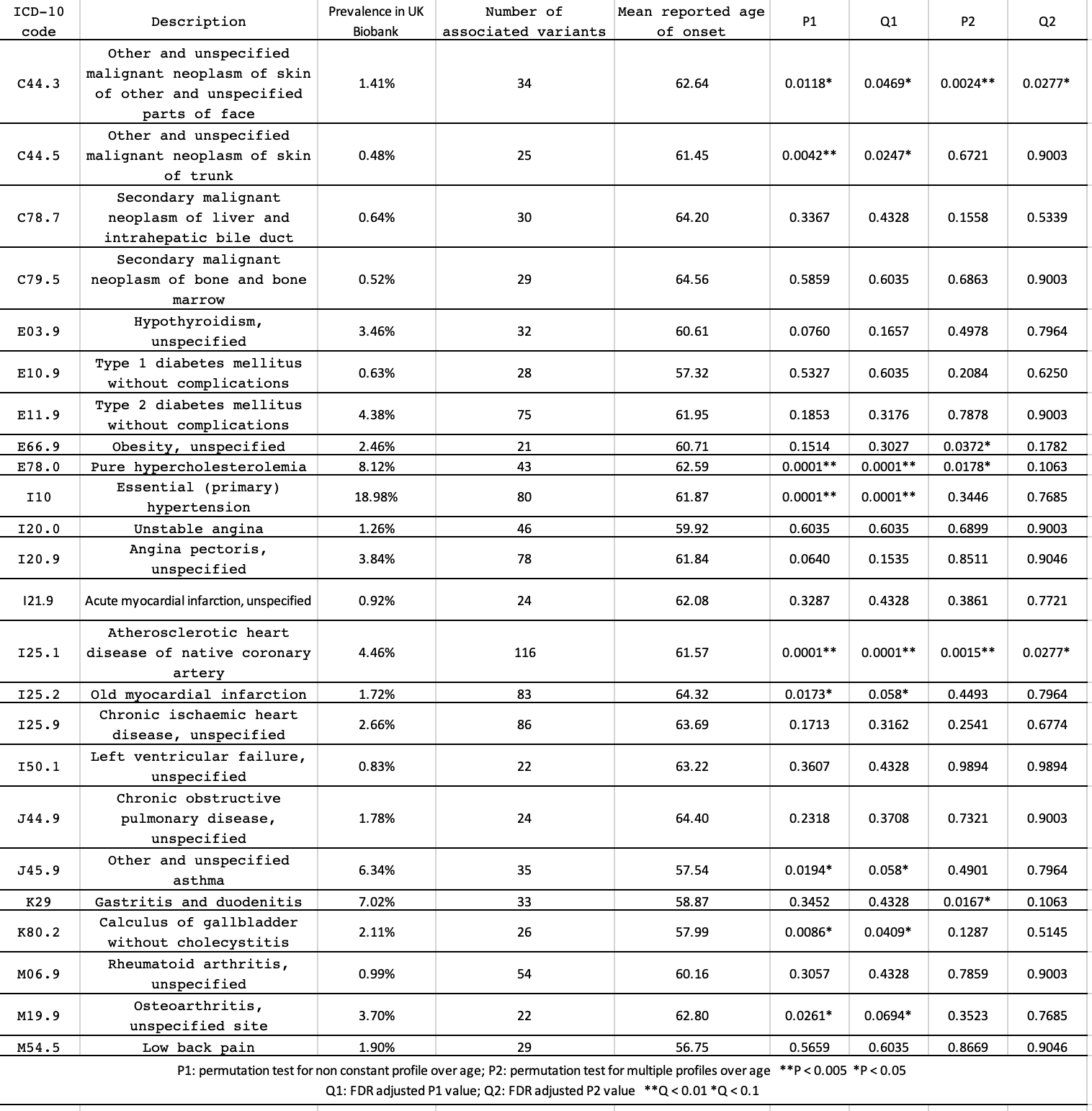

Supplement: S1 Table — Summary of ICD-10 disease codes analysed and evidence for age-varying effect sizes and number of age-profile classes, fitted with a univariable model and quadratic polynomial. Details are for Table 1. (PNG) [file pgen.1009723.s016.png]

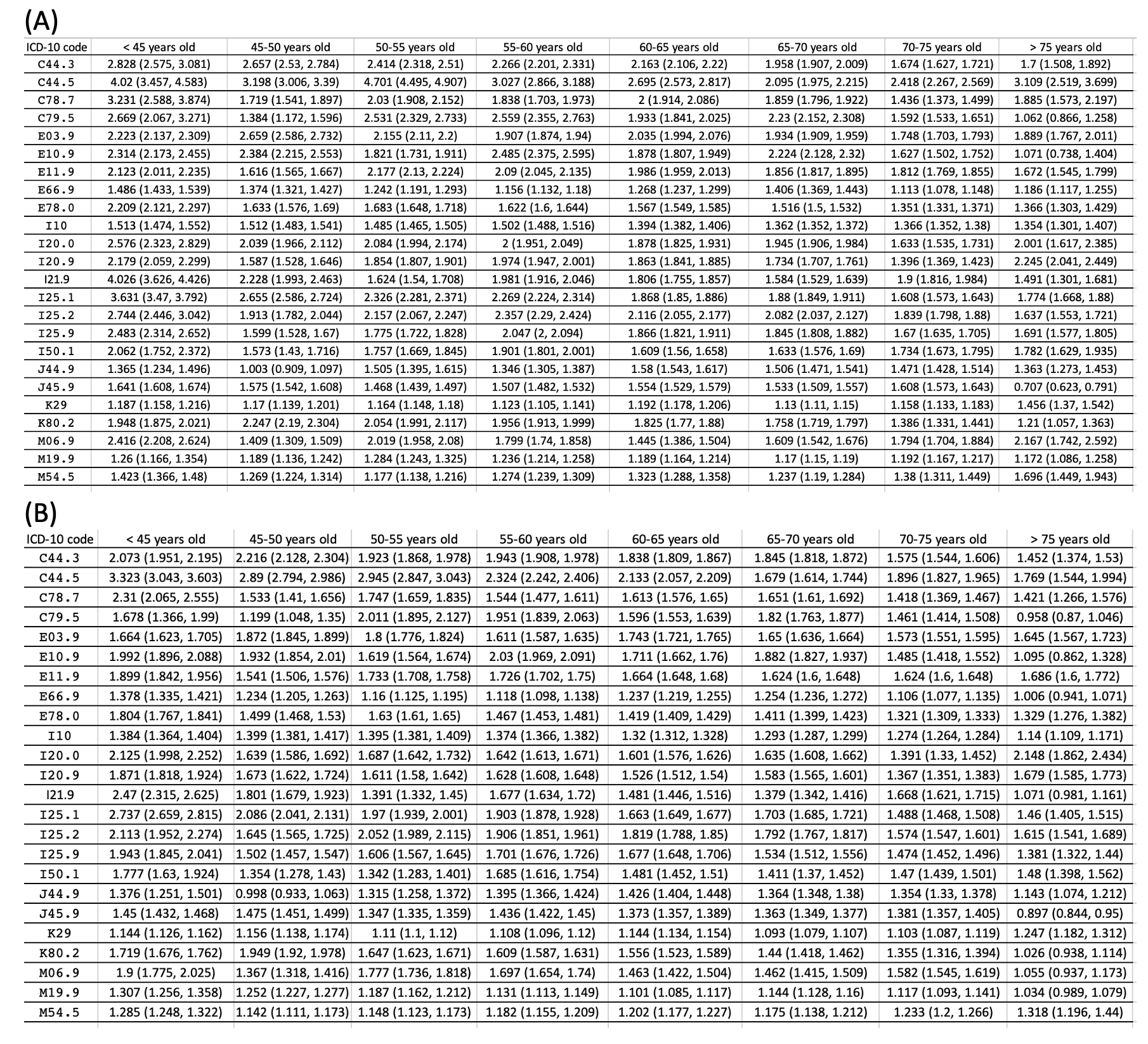

Supplement: S2 Table — (A) Mean odds ratio of the 90th percentile GRS over the population average. (B) Mean odds ratios of the 80th percentile GRS over the population average. Parentheses contain the 95% confidence intervals of the mean. (PNG) [file pgen.1009723.s017.png]

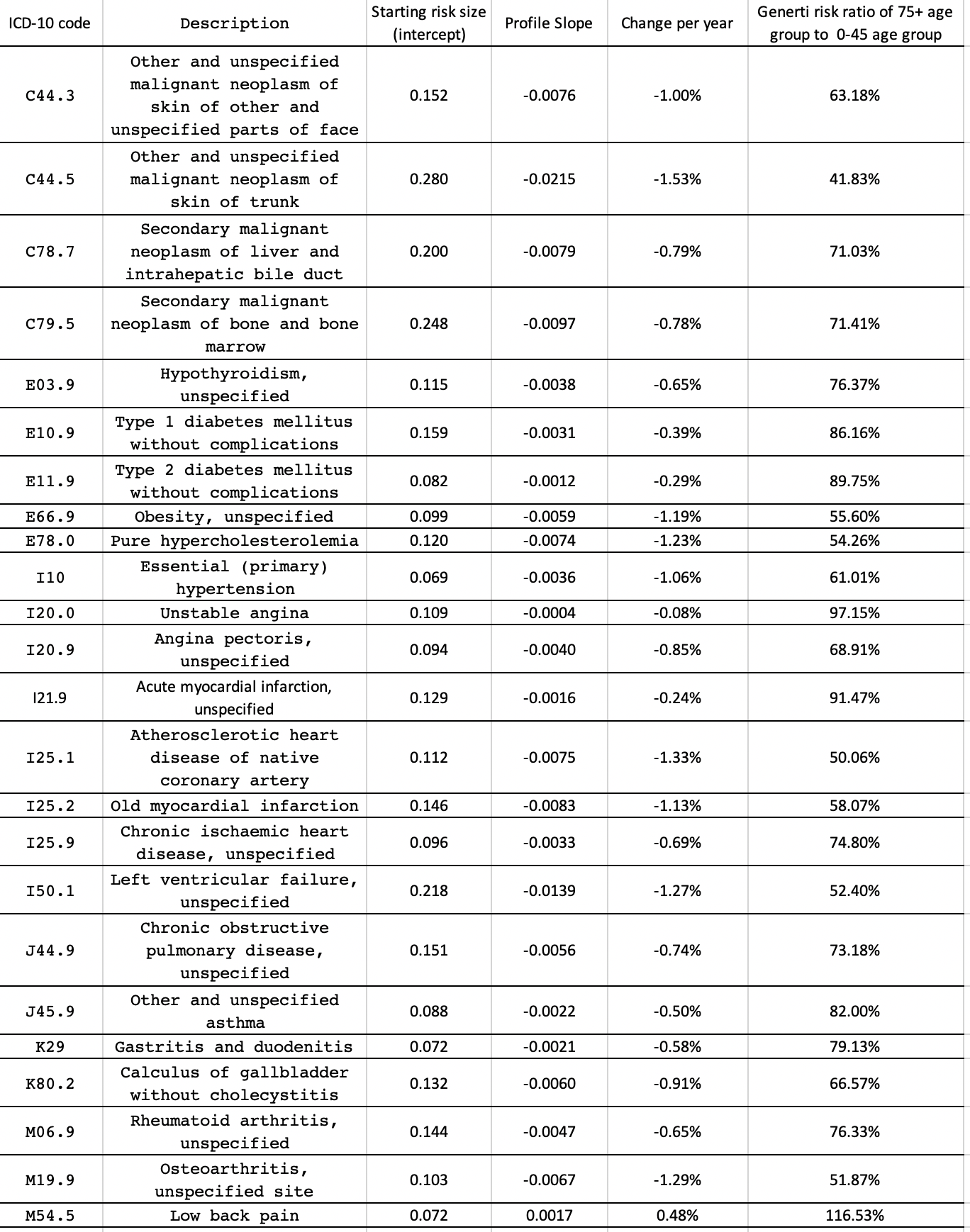

Supplement: S3 Table — Summary of changes in genetic risk contributions from before 45 years old to after 75 years old, when risk profiles are fitted using a linear model. (PNG) [file pgen.1009723.s018.png]

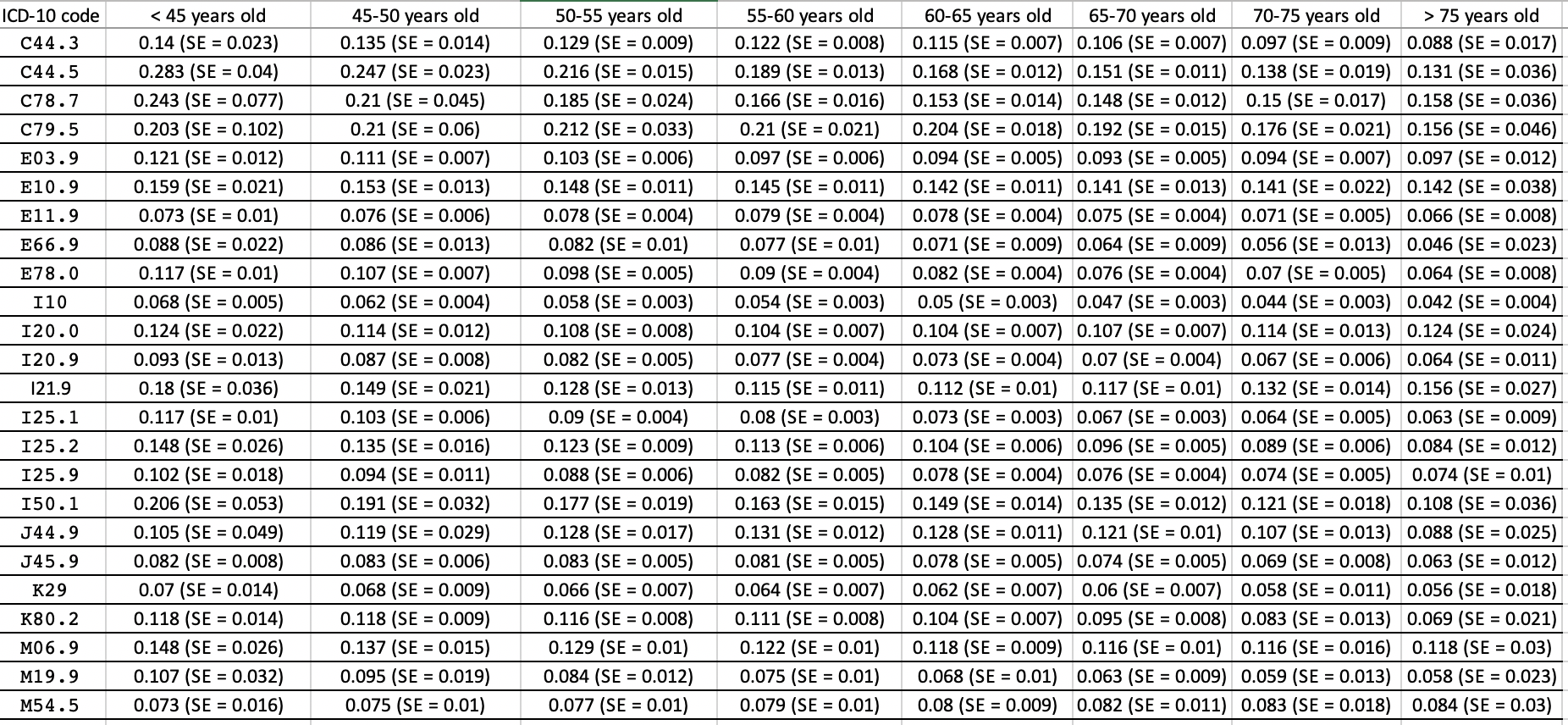

Supplement: S4 Table — Posterior mean risk profiles for all diseases analysed here, fitted with a single quadratic polynomial. Standard errors are also provided. Values are the mean effect size within the age interval for individual variants. (PNG) [file pgen.1009723.s019.png]

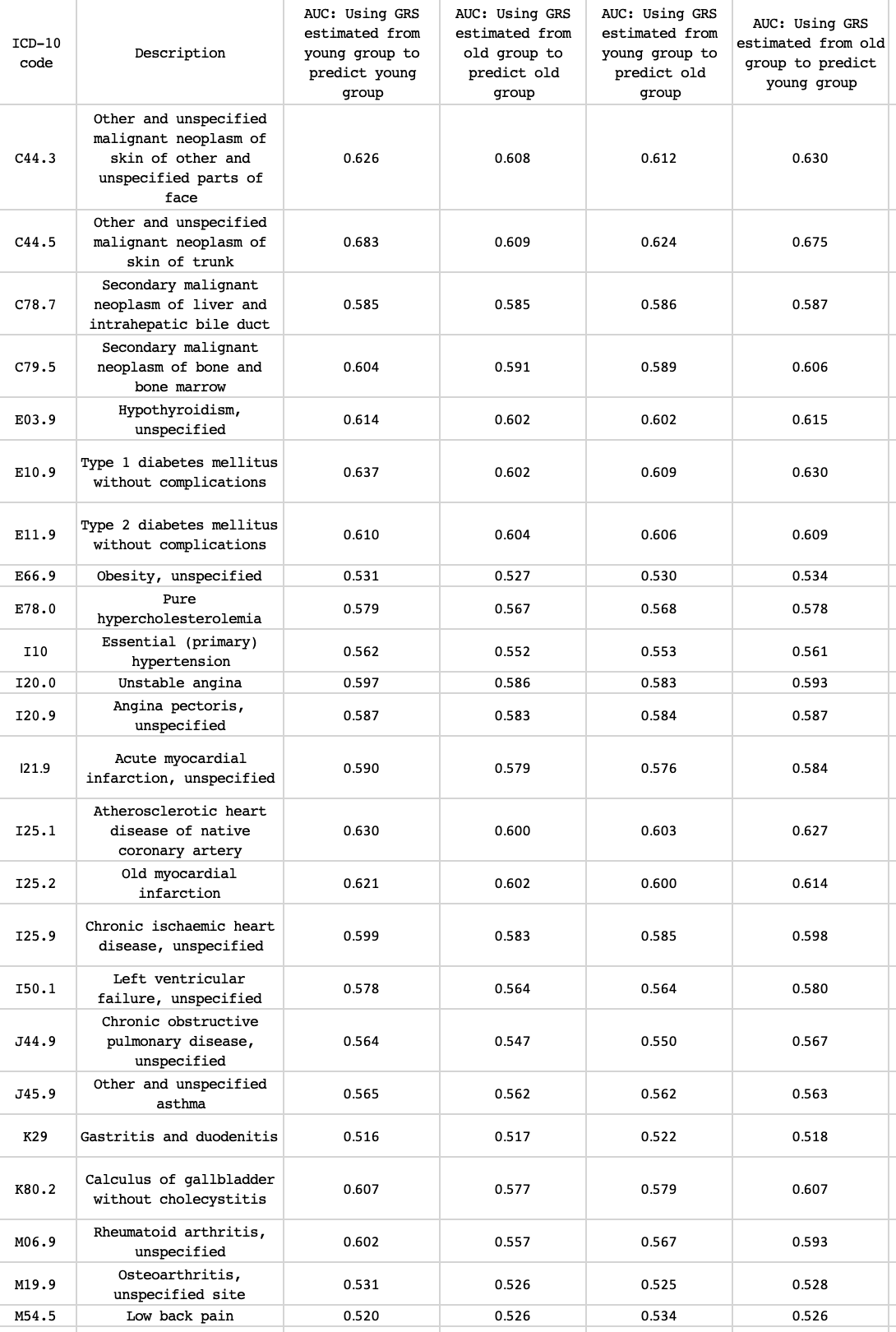

Supplement: S5 Table — The population is divided into a younger group and an older group, where GRS is estimated in the training set from one group and receiver-operating curves (ROCs) are computed from the testing set in one group. By the combining age assignments for the training and testing sets there are four conditions: GRS and ROC are both computed from the younger (third column) or older group (fourth column); GRS is computed from the younger group and ROC is computed using the older group (fifth column) and the other way around (sixth column). Regardless of whether the training data and testing data are from the same age group, we use 80% of the sample as the training set and 20% as the testing set to match the sample sizes of each condition. (PNG) [file pgen.1009723.s020.png]

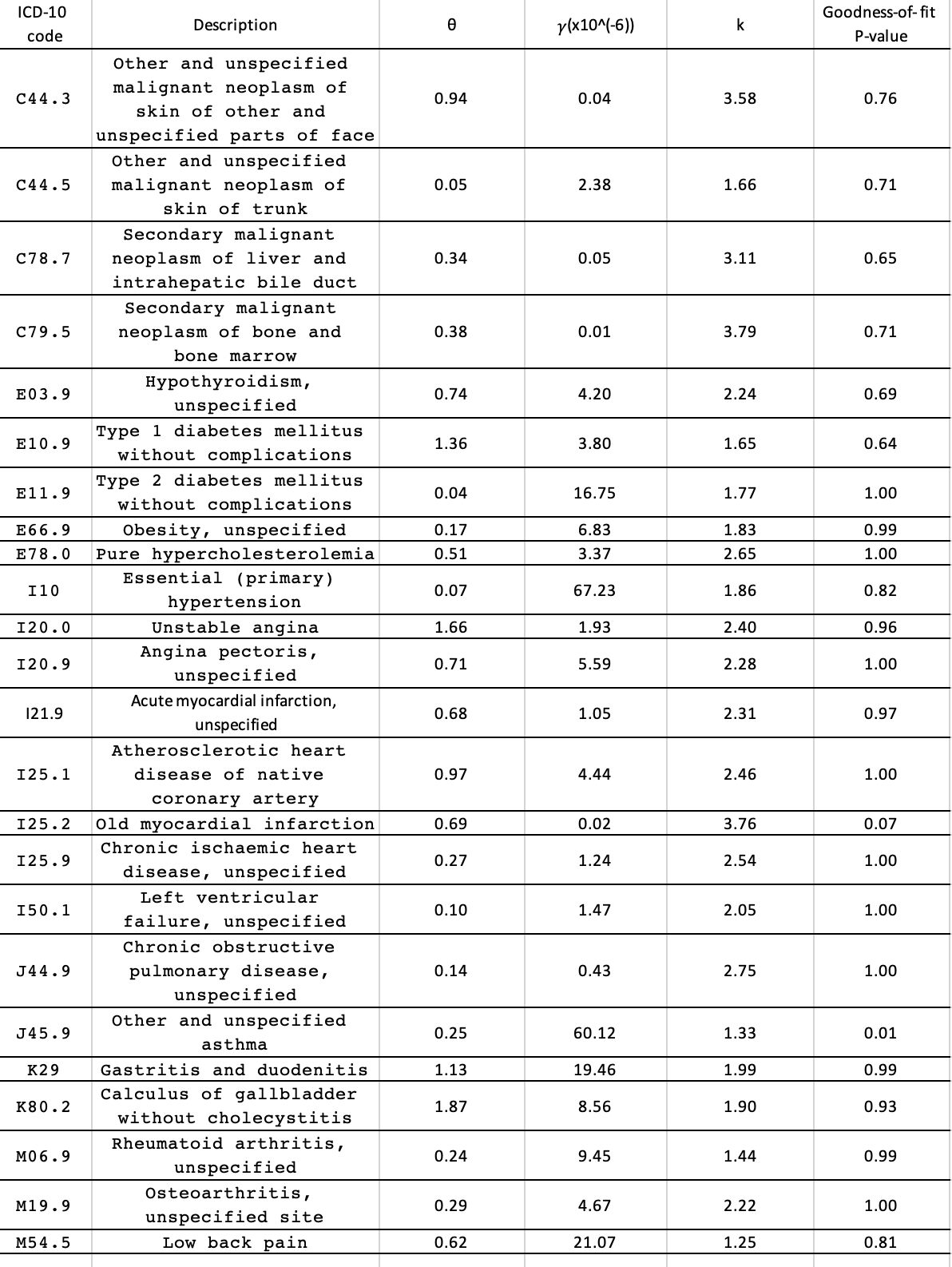

Supplement: S6 Table — The fitted model has a hazard rate of hi = ui γtk, where ui ~ Gamma(shape = 1/θ, scale = θ). (PNG) [file pgen.1009723.s021.png]

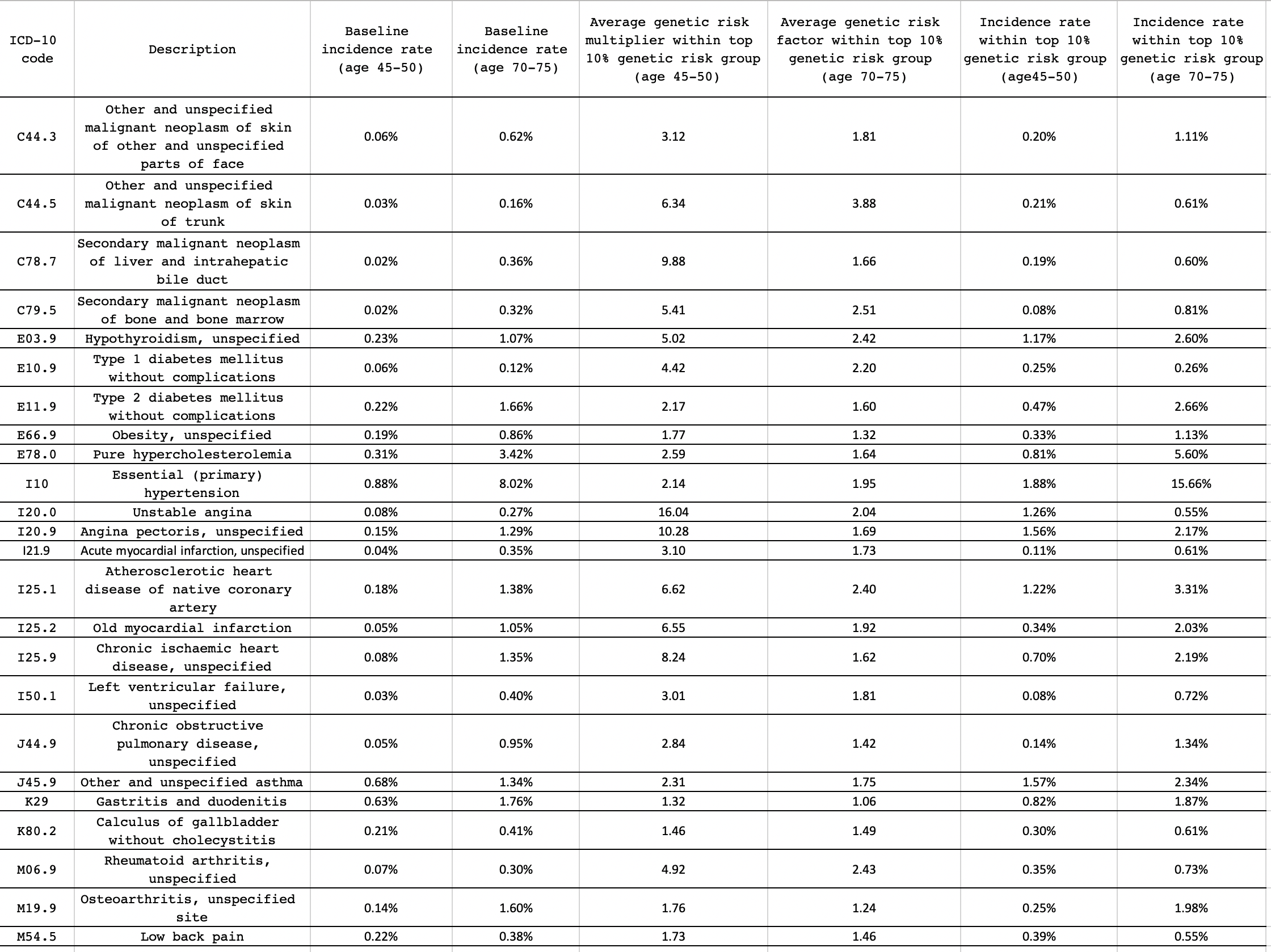

Supplement: S7 Table — Comparison of baseline hazard rate (population-level risk), genetic risk factor effect size and absolute hazard rate (baseline hazard multiplied by genetic risk factor) for an early age group and a late age group across the diseases studied here. The genetic risk factor and absolute hazard are computed from the group with the highest decile of genetic risk. (PNG) [file pgen.1009723.s022.png]
