# Supplementary material for: The impact of age on genetic risk for common diseases
Source: PLoS Genet. 2021 Aug 26;17(8):e1009723. doi: 10.1371/journal.pgen.1009723 (PMC8389405; doi:10.1371/journal.pgen.1009723)
Supplement: Attachment — Submitted filename: point to point response to comments.docx [file pgen.1009723.s023.docx]

**From the Editor:**

In addition to addressing comments of the reviewers, please explain and justify the limitation of analyses to persons of British Isles ancestry. Please provide parallel analyses in supplementary materials that include all UK Biobank subjects regardless of ancestry, with any desired adjustments explained and justified, and describe them briefly in the main body of the paper along with description and discussion of similarities and differences from the British Isles subgroup analysis. Stratified analyses by ancestral groups may also be included and commented upon at your discretion.

The British Isles ethnicity subgroup is the largest within the UK Biobank (80% of the cohort) and by focusing analyses on this group we aimed to reduce the potential for confounding that can occur by combining data from across groups with different patterns of genetic variation, exposure and patterns of interaction with healthcare. However, we recognise the weakness of this choice and hence have also repeated our analyses both at the level of the entire UK Biobank (controlling for stratification) and within the two other largest ethnic groups within the cohort (individuals self-identifying as of Black or Black British and Asian). The results are now included in the paper and discussed. We observed only one notable difference in the stratified results, whereby J45.9 “asthma” shows an apparent increase in genetic risk over age within the Black or Black British subgroup (P = 0.006).

Changes: manuscript line 102-114, 216-221; Fig S6; Fig S7; Supplementary methods section 1.2.

Please also add a limitations section to the Discussion.

We have added a paragraph of the limitations of the current work within the discussion. This includes a discussion of the issue of potential heterogeneity among subgroups.

Changes: manuscript line 357-369.

## **Reviewer 1**

Jiang et al detail a new method to determine the non-linear relationship between age and genetic effects, using the UK Biobank as the applied dataset. The systematic identification of unique genetic profiles that differ by age is a meaningful contribution to the literature, as is the elegant exploration of different factors (frailty, bias) that could influence these relationships. The manuscript as it is currently framed is somewhat in between the presentation of a new method and the application of the new method to better understand genetic risk in the UKBB. It would be beneficial to the manuscript to do a little bit of a tweaking to help the reader understand how this method could be used in their own workflows. This would require showing how the identification of these profiles is important when estimate genetic risk. While this would require additional analyses, I think it would make the manuscript more impactful. In short, when I read this paper I’d like to have a figure to point to and say “Ah! If I hadn’t used this method to find non-linear relationships by age, I would have created a model and given back false risk estimates to my patients!” A few major and minor points are outlined below.

We thank the reviewer for their supportive comments. In response to the points about demonstrating the impact of the findings we have included an additional analysis looking at the direct impact of age-varying genetic relative risk on prediction accuracy. We find that prediction in younger individuals (as measured by AUC) is considerably higher than in older individuals. The implication is that a risk score that is not modulated by age could both underestimate the risk in young individuals and overestimate it in older individuals.

Major points.

• The authors estimate age-specific effects for each SNP within each interval. However, the original set of variants were selected according to a previously published TreeWAS approach, which was conducted across the entire UK biobank sample. Is this original selection weighted towards younger individuals, and therefore younger cases? This may be outside of the scope of this manuscript, but it would be interesting to know how much of the decrease in genetic risk is due to selection bias from this original analysis (i.e. do different loci contribute to risk as we age?)

This is a good point. To address this, for two diseases we repeated the primary analyses using genetic variants reported by external studies and captured in the GWAS catalog. We chose two of the ICD10 codes in the paper: I10 “primary (essential) hypertension” is the most prevalent code among our 24 disorders; I25.1 “atherosclerotic heart disease” has the most associations. In GWAS Catalog, the trait “hypertension” has 733 associations from 90 studies. Among these associations, 653 are unique and 191 of them are genotyped (missing rate < 10%) in the UK Biobank (only 20 of these variants intersect with the variants detected by TreeWas). The trait “coronary artery disease” has 1,783 associations from 119 studies. 1,319 variants from these studies are unique and 290 of them are genotyped in the UK Biobank (only 25 of these variants intersect with TreeWas results). We inferred the genetic risk profiles for these variants using the same procedure, and found similar patterns (but of different magnitude) compared to those using TreeWas detected variant sets.

Changes: Fig S8; Manuscript line 223-228; Supplementary methods Section 1.2

• Throughout the manuscript it would be beneficial to include the actual numbers, instead of referring to trends, especially when the numerical results are in the supplemental tables (or require going through another dense table). For example, page 8 lines 120-123 include phrases such as “dramatic decrease” and “gradual decline”. It would help to include some numbers to help contextualize these phrases.

The changes in odds ratio (as well as bootstrap confidence interval) were added to the text. We also provided another two tables containing all the mean and 95% confidence intervals for the odds ratio within 90th and 80th percentile GRS in Table S2.

Changes: Manuscript line 131-140; Add Table S2

• Figure 2 is very helpful in understanding the method, as well as the framework of the manuscript. It would be even better to include a panel within Figure 2 that refers to the “Age-profiles for genetic risk scores” to contrast the different approaches. (Then switch Figure 2 to Figure 1 and push currently Figure 1 down.)

We updated Figure 2 with a schematic workflow (upper panel) explaining how we computed genetic risk scores and used the set of coefficients to compute odds ratio of high GRS within each age interval. The order of figures are rearranged as suggested.

Changes: Fig 2; Caption of Fig 2; order of Fig 1 and Fig 2.

• The new method detailed is to determine non-linear relationships between age and genetic risk. However, the majority of the results sections do not fully explore the non-linear changes in risk. For example, in the “Application to common diseases in the UK Biobank” section (pages 10-11), the authors compare risk at 45 and 75. However, there are some results in which risk is largely unchanged up until a certain age (Figure 4F), which would support a non-linear relationship and warrant expansion. (I basically am saying that you are underselling your method and findings. It would be great to have more on these non-linear trends!)

We were aware that a simple linear model might not capture the age-dependency in genetic risk well enough, which is the reason we chose to present profiles fitted with a quadratic polynomial. Figure S3 showed how we chose the complexity of the model, where we compared linear, polynomial and spline models for fitting the risk profiles. However, in our permutation tests, we did not find any diseases that are significant when fitting with a non-linear model, but not when fitting a linear model. Therefore, we concluded our primary finding is that genetic risk has a stronger effect at early age while not emphasising the non-linear age-dependency. We did touch on the nonlinearity by mentioning that the forms of age-dependency vary across diseases. Large though the UK Biobank is, more power to investigate non-linearity will come from focused disease cohorts.

• The authors stop one step short of their stated motivation, which is to see how risk prediction can be improved by allowing factors to be modulated by context. It would be great to include this in the manuscript. This method clearly shows how you can identify risk profiles that change over age in a non-linear fashion. You could compare a standard PRS approach, in which age is added into the model with one set of effect weights for the PRS, to an approach in which effect weights are changed based on age bucket. I imagine the model fit would improve, especially for older populations, for those with non-linear trends with this approach compared to the more standard adding of age as an independent linear risk factor. In short, going one step further and showing the consequences of ignoring these relationships versus addressing them when conveying an individual’s risk (ranking) would be important.

To address this point we have looked explicitly at the ability of GRS estimated - in either the younger or older half of the population - to predict risk (in both younger and older groups, so four analyses for each disease). In line with the results already presented, we find consistently higher AUCs in the younger group and that the younger group typically performs better at predicting risk in the older group, than the GRS estimated in the older group (18/24 cases) as expected if the signal is shared between groups but the signal to noise ratio varies. These results are included as Fig S9 and Table S5.

Changes: Added Figure S9 and Table S5 show the comparison of AUCs computed from age-stratified GRS for each trait; Manuscript line 233-249; In Section 2.5 of Supplementary Methods, we described the methods we used for computing the AUCs for each disease.

• It would be helpful to provide some discussion about specific UKBB results, such as why would there be nonlinear relationships between certain ICD-10 codes, genetic risk, and age? Is there any literature about these specific outcomes that would explain some of this? Biological plausibility would add another layer of impact.

Although we explored various potential biological explanations for the phenomena described here, we did not arrive at any firm conclusions. Rather than include speculation, we note this as a limitation of the current work. We hope that these results will encourage future research in the area.

Changes: manuscript line 335-337

Minor points.

• It’s unclear why the “Data preparation” section is included in the Results. It seems redundant with the Methods section. This paragraph would serve better as a way of introducing the age distribution of your sample, such as how many people fit into the different intervals for each outcome and if they are unbalanced in other ways (i.e. your typical Table 1 for epidemiological studies).

We changed this part to “Data Description” and used it to describe the sex and age distribution of the data set as suggested. We also describe analyses carried out on different groups within the cohort (British Isles ancestry, entire cohort and ethnic subgroups), diseases defined by other ontology systems and external variant sets.

Changes: Manuscript line102-114

• The second ICD-10 in Table 1 technically has a prevalence of 0.48%, which is lower than the 0.5% in the methods section (and first results section paragraph).

A good catch. The 0.5% threshold for filtering codes was based on the prevalence within all the hospital episode statistics codes, where the same code could be repeatedly recorded for one individual. The “Prevalence in UK Biobank” in the table refers to the population prevalence (i.e. the proportion of the population that have at least one record of the ICD-10 code). We have added a clarification in the caption of Table 1.

Changes: Footnote to Table 1.

• Please include confidence intervals for the odds ratio estimates presented throughout the manuscript. (For example, page 8 line119)

We have added confidence intervals for each odds ratio (or more precisely, bootstrap confidence interval as we have drawn 20 bootstrap divisions of samples for cross validation). We also provided additional tables that contained the mean and 95% confidence intervals for the odds ratio within 90th and 80th percentile GRS in Table S2.

Changes: manuscript line 131-140; Add new Table S2

• Page 12, lines 216-217 say that there is a FDR>5%. Please put actual FDR (how much higher than 5%).

In the simulation studies, the false positive rate increases as the variance of frailty distribution increases and we only reported the observation that FPR > 0.05 since we used P=0.05 as a threshold to reject the Null hypothesis. We now state the minimal value of FPR (0.09) when the variance in risk is greater than 0.1 of the mean.

Changes: manuscript line 265-266

• Typo in Figure 2, panel C under “Estimate genetic risk profiles for all associated SNPs of a specific(e) disease”.

The figure has been updated.

Changes: Fig 2 (now Fig 1)

## **Reviewer 2**

This paper uses UK Biobank data to systematically assess whether hazard ratios for disease-associated SNPs and polygenic risk scores vary by age. A number of papers have already shown that polygenic risk scores have age-dependent odds/hazard ratios or linear regression slopes for particular diseases and traits: age-specific effects have been observed for breast cancer [Mavaddat PMID:30554720 and PMID:25855707], prostate cancer [Conti PMID:33398198], breast and cervical cancer [Kachuri PMID:33247094], weight [Khera PMID:31002795], BMI [Mostafavi PMID:31999256], et cetera. This paper makes a contribution to that literature by focusing on age interactions, examining them in a systematic way across 24 common diseases in a large population-based cohort, and using appropriate methods for time-to-event data.

We thank the reviewer for their positive feedback.

Weaknesses of the paper include:

--imprecise language ("biased"/"unbiased" without clarity as to the target parameter to be estimated; references to "effect" without specifying the relevant effect measure [e.g. age-specific hazard ratio conditional on the unobserved frailty vs. age-specific hazard ratio marginal over the unobserved frailty vs. age-specific risk difference marginal over the unobserved frailty);

--lack of clarity in the description of the methods;

--and a tendency to overstate the implications of these findings both in terms of prediction (which will depend on epidemiologic and clinical context) and in terms of underlying etiology (finding that a model with age-constant genetic hazard ratios or age-dependent hazard ratios or an accelerated failure time model or a liability threshold model is a better empirical fit does not necessarily imply that a gene "acts biologically" at one stage of life or another: e.g. under a "two-hit" process for carcinogenesis, I can come up with two plausible mechanisms that would lead to increasing hazard ratios with age, one where genes only act early in life, and one where they only act later).

We thank the reviewer for taking the time to go through the paper carefully and identify where there was insufficient clarity, accuracy or precision in terms of claims. We have adopted nearly all of their suggestions, removing the more sweeping statements and improving the precision and appropriateness of the language.

Specific comments follow.

Line 60-61: "... though see [6]." Spell out the specific reservations described in reference [6].

We have updated the manuscript to include “one study has suggested that PRS does not provide additional prediction power over clinical risk factors for coronary heart disease”

Changes: Manuscript line 63-64

Line 62: "... has received relatively little attention..." Hm. Not how I would characterize the part of the elephant I'm familiar with. I do think it's fair to say the extent and impact of age-specific effects has not been thoroughly or systematically explored.

We have replaced “has received relatively little attention” with “not been fully explored”.

Changes: Manuscript line 66

Line 63-63: "One aspect... is the role of age in modulating effects. Several studies have identified variants that influence age-at-onset... Similarly individuals with high PRS tend to have earlier age-at-onset..." The first sentence in this paragraph and the next 1.5 sentences are apples and oranges. Under a simple age-constant genetic hazard ratio model with no unmeasured risk factors, genotype/PRS will be associated with earlier age at onset. Association with age at onset in itself need not imply age modulates genetic effects. The clause "... genetic analyses of quantitative traits... whose effect size changes with age" is more relevant to the paragraph's topic sentence. And see the examples above of disease traits where age-specific hazard/odds ratios have been observed. Also pathogenic variants BRCA1/2 have well-known and large gradients in relative risks as a function of age.

We have updated these sentences to include the literatures listed above as references, and mentioned that studies have shown PRS have age-dependent prediction power for several diseases. We removed the reference to genetic affect age-at-onset for diseases.

Changes: Manuscript line 67-70

Line 74: "...hidden risk factors can act to apparently reduce risk over time..." Not risk. Relative risk.

We were indeed using ‘risk’ to mean ‘relative risk’ throughout. We hope that the revised manuscript is now clear on this point.

Changes: Manuscript line 78; throughout the manuscript, we replaced 24 “risk” with “relative risk”

Lines 74-75: "... a phenomenon typically referred to as frailty in the epidemiologic literature." Hm. I don't know that confounding between measured and unmeasured risk factors induced by survivor bias is referred to as frailty--frailty refers to a set of models accounting for unmeasured risk factors. Anyway, safe to cut this clause. You introduce frailty and how it can lead to differences in age-specific hazard ratios (marginal over frailty) later.

We have removed this clause.

Changes: Manuscript line 78

Lines 90-94: I like the analyses that quantify the anticipated drop off in the age-specific marginal hazard rates due to unmeasured risk factors. But the last sentence is too strong. First, it's possible some frailty model that you did not explore could account for some of the effects. Second, and more importantly, it's really hard to argue from relative goodness of fit to different statistical models for "interaction" to underlying biological mechanisms (Siemiatycki PMID:7327838, Thompson PMID:1999681). E.g. a biological model where genes only act during puberty to set ones later susceptibility to breast or prostate cancer would be consistent with an age-constant relative risk model, as would a biological model where the genes are acting constantly across ages. Just tone this sentence down a little, and be more specific--e.g. "Our observations suggest that genetic relative risks (conditional on all other risk factors) vary with age" is on more solid ground than trying to tie that statistical phenomenon to an underlying biologic process. Especially when talking about so many different diseases, with distinct etiologies.

We have modified this sentence using the suggested language: “our observations indicate that genetic relative risks (conditional on all other risk factors) vary with age.”

Changes: Manuscript line 95-96

Line 106: "We used eight age intervals..." This seems out of place here. Plus not accurate, right? <45 and >75 are not five-year intervals. Can probably just cut this sentence.

This sentence has been removed.

Changes: Manuscript Line 102-103

Figure 1 and throughout: The authors never specify what they mean by odds/hazard ratios for the 80th or 90th %iles. I assume that means top 20% resp. 10% versus the bottom 80% resp. 90%. If so, those are hard measures to interpret--they are driven in no small part by the folks in the lower (protective) %iles. Clinically and intuitively, the risk relative to population average is of most interest. Given this, and given the somewhat distracting duplication of every curve (80% and 90%), I suggest presenting the odds ratio/hazards ratio per standard deviation of the PRS. That also makes these results more directly comparable to other results in the literature, which report per-sd effects.

We computed the ratio of odds in the 90th and 80th percentiles of the GRS distribution versus the odds in the population. We added “versus the whole population” to clarify how we computed the odds ratio in the Methods section “Estimating age-dependency of genetic risk score in prediction”. Using risk in high percentiles of PRS as a risk factor for outcome prediction is not rare in literature, see Table 2 in PMID: 33398198, Figure 1 in PMID: 29789686. The reason for showing two curves is to exclude the possibility of selection bias in the group with highest GRS. The multivariable nature of GRS implies that higher percentile corresponds to stronger prediction power. Therefore, we should see that the odds ratio of the 90th percentile lies above and has a similar pattern to the 80th percentile (which indeed we do).

Changes: Manuscript line 443-444.

Lines 129-134, Methods, Supplementary methods, et cetera. I don't understand what was done here. Are the authors (a) creating case-control sets from all individuals at risk in the age interval (i.e. excluding those how have previously had an event or been lost to follow up before the start of the interval), and then performing logistic regression? Unconditional? Conditional on matching factors? Or are they (b) performing Cox proportional hazards regressions separately for each age interval (as in the Anderson-Gill model, allowing for age-interval-specific genetic hazard ratios)? Note: these two approaches differ in how they handle age-at-event within age intervals. In a Cox regression, as case who has an age later than the index case but still in the same interval would be treated as a control for the index case. In a logistic regression, none of the cases that occur in the interval would be treated as a control. For most diseases and most age intervals (the five year intervals), the two approaches shouldn't differ much. But for more common diseases and for the longer age intervals (<45 and >75), they might.

Our primary analyses used a Cox proportional hazard model for each age interval which is (b), as stated in Methods Subsection “estimating age-specific effects of genetic risk factor” (Line 452-455) and Supplementary Methods Section 1.2.3. However, we also applied logistic regression in two prediction analyses in the paper: when constructing GRS for risk prediction for Fig 2; when computing GRS for younger and older groups in Fig S9. The updated Figure 1 separates and compares two approaches, which were applied in parallel to each disease.

Changes: Fig 1 where we present additional workflow to help the reader understand there are two methods applied.

Other Qs: Why sample controls? Why not use the full risk set? Is this a computational issue or a way of getting fine-scale control of matching factors? If analysis (b) was performed, were the data restricted to cases and sampled controls or was the whole risk set used? If only the sampled controls were used, was the analysis run stratified by matched set (equivalent to conditional logistic regression)?

We used the Cox model with case-control matching to correct - as best possible - for a variety of potential confounders (sex, year of birth, BMI, and 40 genetic PCs). Only the sampled controls were used to perform our analysis (this is not a computational limitation but an analysis choice based on minimising confounding). Cox regression for the matched case-controls was applied within each age interval, though resulting coefficients were fed into a clustering of curves model with smoothing between intervals. Definitions for case and control status are described in Manuscript line 408-428.

Changes: Manuscript line 144-145

Line 138: "univariate or multivariate" I have no idea what this means. Perhaps univariable or multivariable, referring to regressions that do not adjust for measured factors (univariable) or those that do (multivariable)? Marginal (one SNP at a time, averaged over/ignoring the other SNPs) or multivariable (one model with alls SNPs included, a mutually-adjusted model)?

Thanks for pointing this out. All “univariate” has been replaced by “univariable”. All “multivariate” has been substituted by “multivariable”.

Changes: Throughout the manuscript: 11 “multivariates”, 18 “univariate” to multivariable and univariable. Supplementary methods: 3 “multivariates”, 3 “univariate” to multivariable and univariable.

Line 152: Multivariate?

See above.

Line 206: ".... centered on effect size." You can safely cut this, and you should, as it is confusing without writing down what you mean in maths with specifically defined terms. "Effect size" is too vague. (The frailty is centered on the hazard conditional on observed risk factors).

We have deleted this clause.

Changes: manuscript line 254

Figure 5a. What's going on here? Are there four curves being plotted (see esp. second panel)? Red and green, dashed and solid? Uni/multi x empirical not assuming frailty model/assuming frailty model?

We have added legends for Fig 5a to clarify: all purple curves are estimated using univariable methods, with other variants as part of the frailty. Green curves are multivariable estimates and all the variant sets are observed (therefore excluded from frailty). The dashed curves and solid curves were intended to separate clusters, and it is now noted with the legends. These are also clarified in the caption of Fig 5.

Changes: Fig 5a, legends are added to distinguish results from univariable and multivariable models. Caption of Fig 5 line 939-941.

Lines 221-223: "... results were essentially identical under the two approaches..." What two approaches? "Univariate" versus "multivariate"? If so, why should it follow that "genetically-arising" frailty cannot explain the pattern? Again, I am confused as to what "univariate" and "multivariate" mean. If it means "marginal (one SNP at a time, averaged over/ignoring the other SNPs) or multivariable (one model with alls SNPs included, a mutually-adjusted model)," then the conclusion does not follow--I think the main point is that there might be other SNPs besides those included in the risk model that could be driving risk; different ways of modeling the included SNPs won't get at that.

We have changed “multivariate” and “univariate” to “multivariable” and “univariable”. We have modified this sentence to “frailty arising from variants included here cannot explain the pattern”.

Changes: manuscript line 270-272

Lines 252-253: I think you're on pretty firm ground with the last sentence, but suggest replacing "genetic risk" with "genetic relative risk."

We have replaced "genetic risk" with "genetic relative risk". More generally, we have made the focus on relative risk much more evident throughout the manuscript.

Change: line 302

Lines 260-262. Again, I would not say "little attention" has been paid to gene x age/sex/diet/behavior/environment interactions--generally and specifically in the context of risk prediction. Workshops have been held, RFAs have been issued, risk models have considered the inclusion of interaction terms and found some age interactions (although the boost in utility from including such interactions need not be clinically meaningful). A few PMIDs you might find interesting: 24123198 27061572 28978193 28978192 28978190 21284036 33247094 32359158 25380502 27228256 31875139 29458155 22633398. Also, from this journal, PMID:19584936. And I didn't even pubmed Type 2 Diabetes or Coronary Heart Disease.

We have modified the sentence to focus on the specific nature of the work carried out here - namely the role of age in modulating polygenic risk for disease risk prediction.

Changes: line 309-312

Lines 264-266: Best to be specific here. You set out to see if there were differences in genetic relative risks/odds ratios/hazard ratios by age. This is not the same as evaluating the "relevance" of genetic risk information in particular ages, either in terms of prediction (primary and secondary prevention) or in terms of etiology. For example, the per-PRS-standard deviation in genetic relative risk might be 3 for under 45s and only 1.5 for the 60-70s--but if disease incidence before age 45 is extremely rare, the benefits of PRS-targeted interventions might not outweigh the risks, whereas for older ages they might, due to the higher incidence. See also comments above about the challenges inferring biologically meaningful windows from age-specific relative risks.

We have added 'relative' to make this clear and noted the importance of absolute risk in clinical utility.

Changes: line 313

Lines 274-275: "approaches that do not address the selection biases inherent in stratified analysis of longitudinal data." First, only one of these three cited references includes any data on age-specific genetic effects (and that only for one trait). Second, biased relative to what? In the prediction context, I'd argue we're estimating what we want when we estimate the age-specific relative risks marginal over the frailty. After all, we never get to measure the unobserved frailty! This should be noted--good news: if you care about prediction previous results are not useless and not necessarily "biased." If on the other hand you want to estimate the genetic relative risk conditional on the frailty, because you want to use that to make some inferences about underlying etiologic mechanisms, then yeah, the marginal relative risks are biased, and what looks like an age dependent effect may not be "real."

We have removed the reference to bias and only kept the references relating explicitly to the relationship between genetic risk and age.

Changes: line 324

Line 284: "exponentially"--meaningless: everything is tautologically exponentially higher when your are comparing two groups (here 40s to 70s). The ratio is some positive number (other than 1) raised to some power: ergo exponential. Quote a range of observed ratios here (relative risk in 40s over relative risk in 70s from 1.5 to 6 or whatever it is).

We have replaced “exponentially” with “several fold”.

Changes: line 331

Figure 6: What's going on in 6b? It's not clear to me how GxE interaction leads to different colored bits in the histogram. Are you saying 50% of the population is exposed, and they're the ones in the darker color? But how can that be? A mixture of normals is not ever normal, is it? Certainly not in general. Or it's a sum of G and product interaction G*E terms--in which case you should see G and GE contributions over the whole range. Methods lines 524-528 do not clarify this any. Supplementary methods 3.2.2. only gives methods for Figure 6.a. not 6.b.

We revised Figure 6B using the same style Figure 6A and showing the impact of GxE on the variance in liability, how that can change with age and how this combination can lead to frailty. We also updated Supplementary Methods 3.2.2 to explain how we generated the upper and middle panels in Figure 6B. The simulation that generates the lower panel in Fig 6B was described in Supplementary Methods 3.2.1.

Changes: Fig 6 and captions of Fig 6; Supplementary methods 3.2.2

Line 33: The liability threshold model is not any more generalized than the proportional hazards model. The liability threshold model is a specific parametric survival model that implies a specific age-dependent genetic relative hazard function. It may make intuitive sense--perhaps too intuitive--and may provide a better empirical fit--although it's not as convenient to fit)--but I wouldn't call it any more generalized or richer than a proportional hazards model--especially as it is relatively straightforward to modify the Cox partial likelihood to allow for age-dependent relative hazards, as the authors have done here.

We have removed any comparative statements about models and now simply note that the process models provide an interesting route for exploring the observation.

Changes: Manuscript line 353-355

Line 329: What are the parameters on the age and sex distribution that make a disease compatible with your framework? Specifiy.

We have added how we selected codes and added a brief description of the sex distribution of the study population in the main text and the supplementary methods. The codes selected have at least 10 incidences in each age inteval (which excluded I25.8) and should not be sex-related diseases (which excluded C61, D259, N40). Codes that refer to procedures are also excluded (Z37.0, Z95.5 and Z95.1). We have added these details into Supplementary Methods Section 1.1.

Changes: Supplemental Methods Section 1.1; Manuscript line 102-104;

Throughout: "principal components" not "principle components"

We have made this change.

Changes: In manuscript five "principle components" changed to "principal components"; in supplementary methods: four "principle components" changed to "principal components"

## **Reviewer 3**

Jiang and colleagues studied the time varying effect of polygenic risk on 24 common ICD-10 codes with a prevalence of at least 1.5%, within the British ancestry subset of UK Biobank. The set of common disease codes analyzed each had ~10,000 cases in the UK Biobank and the authors matched each case with four controls. They used a proportional hazards model with an interval-based censoring to estimate genetic risk in different age groups. They found age varying risk profiles for 9 diseases including hypertension and atherosclerotic heart disease and for some diseases there were distinct profiles of genetic risk. For most diseases, polygenic risk had the most impact early on with monotonic decrease over time. Using simulation and permutation, the authors proposed several possible models that might explain their observations.

Comments

There really are not ‘24’ diseases but 24 distinct ICD-10 codes. Several related ICD-10 codes were used for various manifestations of the same disease eg atherosclerotic coronary heart disease (MI, unstable angina etc). These ICD codes could have been collapsed into phecodes to increase power to detect the genetic risk variability over time and improve readability.

Following the reviewer’s suggestion, we mapped our ICD-10 codes to PheCodes [(Wu et al. 2019)](https://paperpile.com/c/aWgjhY/BYXU) and found four of the ICD10 codes analysed here could be collapsed to PheCodes. I21.9 and I25.2 both represent “411.20” (myocardial infarction); C44.3 and C44.5 both represent “172.20” (other non-epithelial cancer of skin). We took a union of the cases and variants for ICD10 codes that represent the same phecode and inferred the genetic profiles for these two PheCodes. The inferred profiles for PheCodes are presented in Fig S8. We found qualitatively similar results as in the single code analyses.

Changes: The new analysis is summarised in Fig S8; manuscript: line 110-114, 228-231; supplementary methods section 1.2

Only in 9 of the 24 ‘diseases’ was there variability of genetic risk with age. In the remaining 14, is the lack of variability due to lack of power to detect variability? If not, how do these diseases fit into the framework of genetic risk, liability and frailty outlined by the authors?

As shown in the manuscript, there is substantial variability in inferred profile across diseases and limited evidence in one for an increase with age We therefore do not think that power alone explains this variability - though we have not formally assessed this. Moreover, our analyses indicate that frailty alone cannot explain the decreasing profiles where we see them. In the discussion we have tried to expand our discussion on what might cause the patterns we observe. Moreover, we discuss the lack of power to detect risk variability in other diseases in the limitation part.

Changes: manuscript line 353-355, 360-362.

It would be helpful to know whether estimates of heritability change with age in the various age groups. Along these lines, the impact of family history on the risk of developing disease at different age groups would be of interest.

In effect, our results are equivalent to a change in heritability over age - which we now note in the text. The bigger problem - of looking at inheritance patterns of disease risk factors and the diseases themselves - is an excellent suggestion for future directions, which we now note. The UK Biobank would not enable this, but there are other cohorts where this would be possible.

Changes: manuscript line 339-340, 362-363.

In GWAS, we estimate the effect size within the age group of cases. GWAS participants are typically middle-aged individuals (mean age 62 years). Application of these risk estimates to much younger individuals introduces an element of imprecision. (The same can be said of much older individuals eg 75-80 y). The authors should comment on this.

We agree with this point - indeed the newly included analysis of AUC by age also makes this point. We also now note that targeting future case recruitment in genetic association studies towards those with younger (or conversely older) onset will add value to this area of research.

Changes: added Fig S9; manuscript line 367-369

Several of the diseases are related e.g., Hypertension and Hypercholesterolemia are risk factors for CHD which in turn is the precursor for Left Ventricular Failure. It would be of interest to assess how these associations impact the modeling of genetic risk effect with age.

We agree the comorbidity might underlie the disease progression over age and is an interesting research direction. Realistically, addressing such a question is much better suited to a dedicated cardiovascular cohort with fine-grained longitudinal data. We now briefly discuss how studying combinations of phenotypes is an important next step, though unfortunately we do not have much to add to this at the current stage.

Changes: manuscript line 363-367.

The authors attempt to account for unmeasured risk factors on risk estimates. How about known risk factors? For example, for CHD it would be of interest to assess polygenic risk after adjustment for related covariates (for example hypertension and diabetes).

Our analysis of whether or not frailty can account for the patterns we observe was designed to capture all possible risk factors - whether known or unknown. We agree that looking in detail at specific risk factors is likely to shed light on the underlying mechanisms leading to a dilution of genetic (relative) risk. But we do not think that any such analysis will change the fundamental conclusion. As noted above, our view is that such work is better carried out in a dedicated disease-focused cohort with dense longitudinal measurement.

Changes: manuscript line 363-367.

Do the analyses account for sampling of cases in the case control analysis? Population, age and sex-specific incidences rate could be used to account for how cases and controls were sampled using appropriate sampling weights.

We sampled controls to match factors including sex, year of birth, BMI, and 40 genetic PCs, see second paragraph in “Data preparation” subsection in “Materials and Methods” section. Because we are studying genetic relative risk - rather than absolute risk - we do not believe that we need to adjust our results to reflect population incidence.

The authors partition age between 45-75 by 5-year increments. The partitioning could have been informed by where the baseline hazard rate changes for different diseases. While this may be difficult to do for all diseases, it could be done for one disease, as an example.

We agree that there could be interesting adaptive ways of modelling change points in genetic relative risk. However, as noted above, the data are largely consistent with a linear decrease over age and we likely don't have the data or methods for a more systematic analysis.

Polygenic risk is more strongly associated with disease onset at younger ages; however, the incidence of disease is typically much lower and absolute risk may remain low even though polygenic risk is high. This has implications for clinical use. The authors should comment.

We have added a sentence to note that clinical utility and genetic risk are not equivalent and that other factors - notably incidence - are also key to clinical utility.

Changes: manuscript line 372-377
